# Supplementary material for: Alternative splicing liberates a cryptic cytoplasmic isoform of mitochondrial MECR that antagonizes influenza virus
Source: PLoS Biol. 2022 Dec 21;20(12):e3001934. doi: 10.1371/journal.pbio.3001934 (PMC9815647; doi:10.1371/journal.pbio.3001934)
Supplement: S3 Table — Results from two-way ANOVA followed by Fisher LSD test for data presented in Fig 2. (PDF) [file pbio.3001934.s009.pdf]

Supp. Table 3. Statistical analysis[a] of influenza infection siRNA screens

	Fig. 2a-b[b]		Supp. Fig. 2a-b[c]		Supp Fig. 2c-d[d]	
siRNA target	*P* value	sig[e]	*P* value	sig[e]	*P* value	sig[e]
NXF1	0.003	**	<0.0001	****	<0.0001	****
ABLIM1	0.0932		0.0013	**	0.2818	
ADAR	0.7809		0.2433		0.6918	
ALDH18A1	0.2871		0.1801		0.1555	
ANP32A	0.4756		0.1942		0.5698	
ATP7A	0.7944		<0.0001	****	0.5782	
CCDC47	0.4571		0.4107		0.014	*
EPB41L1	0.251		0.1353		0.5914	
EPDR1	0.1315		<0.0001	****	0.4658	
EWSR1	0.2701		<0.0001	****	0.0986	
FUS	0.4095		0.0205	*	0.9769	
GMPS	0.9369		0.0337	*	0.5055	
GPD2	0.1871		<0.0001	****	0.8706	
HNRNPUL1	0.0319	*	0.0363	*	<0.0001	****
KPNA3	0.3599		0.0067	**	0.4731	
KPNA4	0.0906		<0.0001	****	0.4336	
LGALS3BP	0.7595		0.0003	***	0.4312	
MECR	<0.0001	****	0.0013	**	<0.0001	****
MRTO4	0.9697		0.0045	**	0.9172	
SHROOM2	0.7592		0.0054	**	0.93	
SMCHD1	0.7206		0.1106		0.5983	
SNTG1	0.5135		<0.0001	****	0.3284	
SRSF2	0.459		<0.0001	****	0.2338	
TMEM106B	0.7947		0.0562		<0.0001	****
TPR	0.3615		<0.0001	****	0.047	*
TRIM21	0.4499		0.0007	***	0.1607	

a, Two-way ANOVA followed by Fisher's LSD test
b, A549 multicycle
c, 293T multicycle
d, A549 single cycle
e, ****, $P < 0.0001$; ***, $P < 0.001$; **, $P < 0.01$; *, $P < 0.05$
