# Supplementary material for: Alternative splicing liberates a cryptic cytoplasmic isoform of mitochondrial MECR that antagonizes influenza virus
Source: PLoS Biol. 2022 Dec 21;20(12):e3001934. doi: 10.1371/journal.pbio.3001934 (PMC9815647; doi:10.1371/journal.pbio.3001934)
Supplement: S4 Table — Sequences used to build phylogenetic tree in Fig 6E. (PDF) [file pbio.3001934.s010.pdf]

Supp. Table 4. Accession numbers for MECR homologs

Common Name	Species	MECR[a]	cMECR[a]
Human	Homo sapiens	NP_057095.4	NP_001336643.1
Chimpanzee	Pan paniscus	XP_003828155.1	XP_034815583.1
Gorilla	Gorilla gorilla gorilla	XP_004025368.3	XP_018869404.1
Rhesus macaque	Macaca mulatta	NP_001248098.1	XP_014988731.1
Pale spear-nosed bat	Phyllostomus discolor	XP_028368816.1	
American bison	Bison bison bison	XP_010828514.1	
Cow	Bos taurus	NP_858055.1	XP_024855139.1
Pig	Sus scrofa	NP_001231011.1	XP_020949000.1
Horse	Equus caballus	XP_001503984.3	
Beluga whale	Delphinapterus leucas	XP_022407680.2	
Camel	Camelus ferus	XP_006175527.1	XP_032351592.1
Alpaca	Vicugna pacos	XP_031539466.1	
Mouse	Mus musculus	NP_079573.2	XP_036020031.1
Chicken	Gallus gallus	XP_024998883.1	
Goose	Anser cygnoides domesticus	XP_013050980.1	XP_013050980.1
Duck	Anas platyrhynchos	XP_027299737.1	
Saker falcon	Falco cherrug	XP_027668811.1	XP_005433096.1
Golden eagle	Aquila chrysaetos chrysaetos	XP_029895600.1	XP_029895602.1
Alligator	Alligator mississippiensis	KYO48103.1	XP_019344297.1
Salmon	Salmo salar	XP_014055905.1	
Trout	Salmo trutta	XP_029592657.1	
Zebrafish	Danio rerio	AAI53449.1	
Frog	Xenopus tropicalis	NP_001016371.1	XP_012812419.1
Slime mold	Dictyostelium fasciculatum	XP_004362847.1	
Nematode	Trichinella spiralis	XP_003380885.1	
Baker's yeast	Saccharomyces cerevisiae	NP_009582.1	

a, NCBI Reference Sequence ID
