# Supplementary material for: Alternative splicing liberates a cryptic cytoplasmic isoform of mitochondrial MECR that antagonizes influenza virus
Source: PLoS Biol. 2022 Dec 21;20(12):e3001934. doi: 10.1371/journal.pbio.3001934 (PMC9815647; doi:10.1371/journal.pbio.3001934)

Supporting Information Raw Images: Uncropped blots for Figures 1B, 2C, 3ACD, 4DE, 5BDE, 6BCF, and Supplementary Figure S5BDF. Areas of interest used in figures are outlined with a red box. Experimental approach, antibody (where relevant), detection strategy and equipment are indicated for each image. Sizes are shown corresponding to molecular weight markers for proteins or 100 bp ladders for PCR products. Lanes not used in the figure are marked with “X”.

Fig. 1b (top)

K627, Western blot, α-FLAG, chemiluminescence, Odyssey Fc

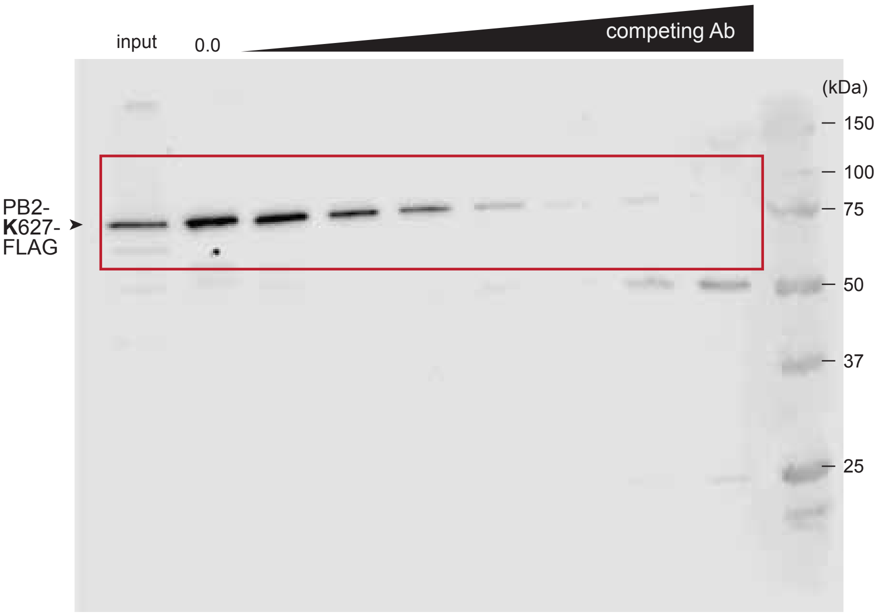

Fig. 1b (middle)

E627, Western blot, α-FLAG, chemiluminescence, Odyssey Fc

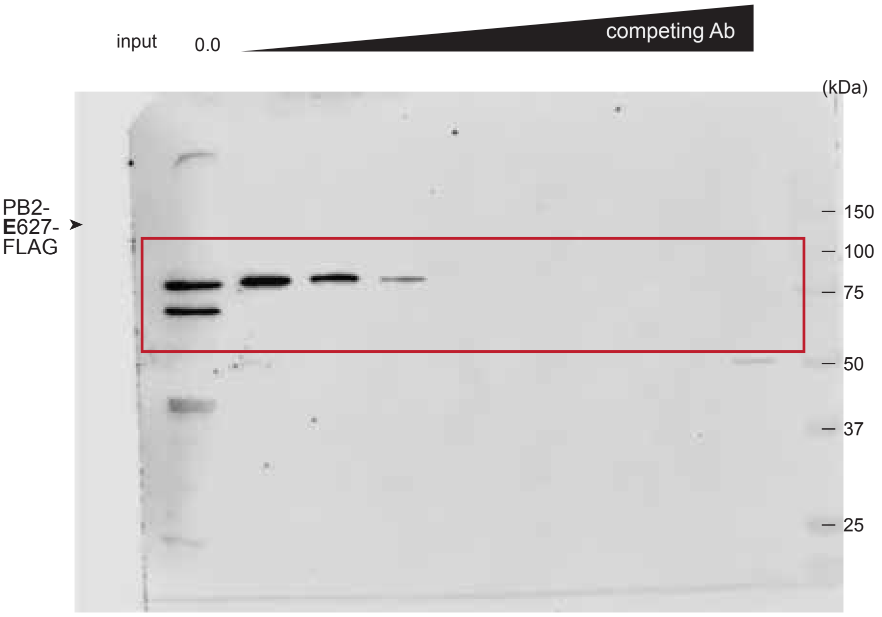

Fig. 1b (bottom)

K627, SDS-PAGE, silver stain, PC photo scanner

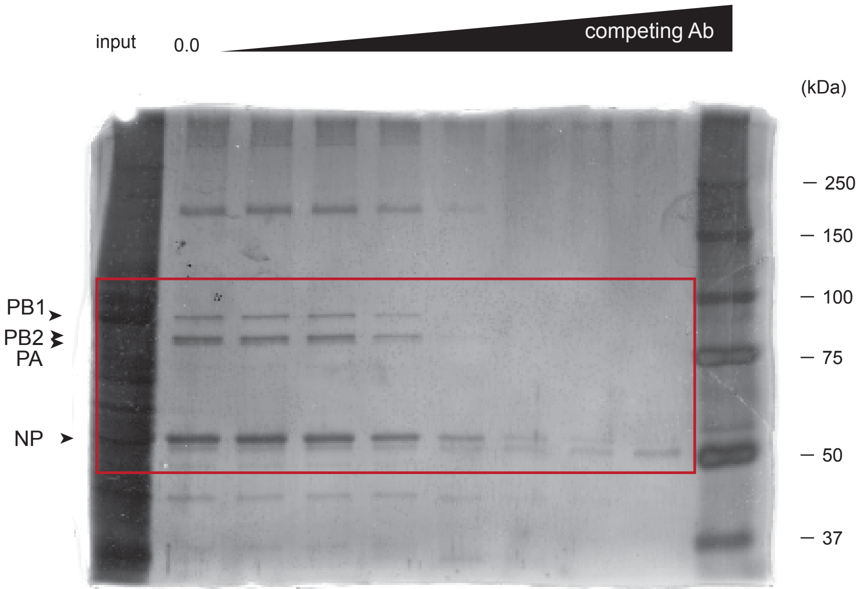

Fig. 2c (left)

hnRNPUL1, Western blot, α-UL1, chemiluminescence, Odyssey Fc

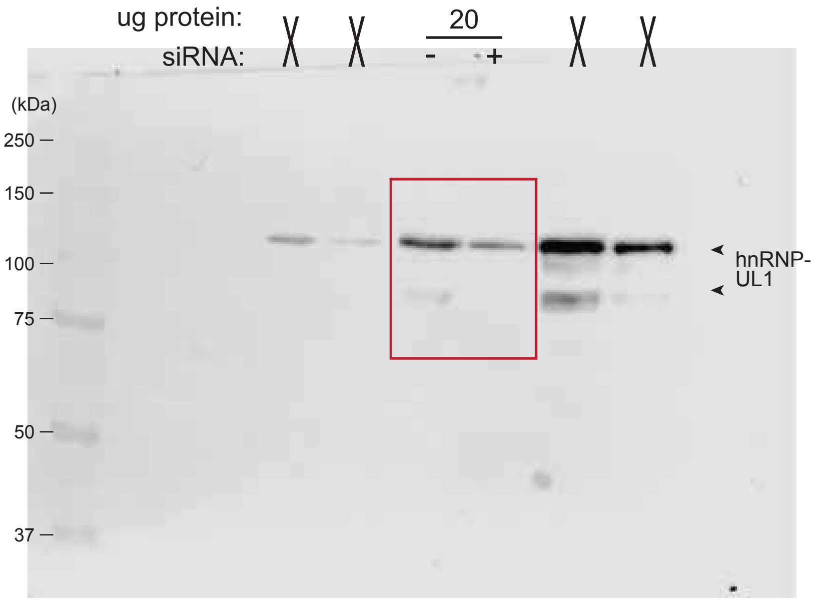

Fig. 2c (right)

MECR, Western blot, α-MECR, chemiluminescence, Odyssey Fc

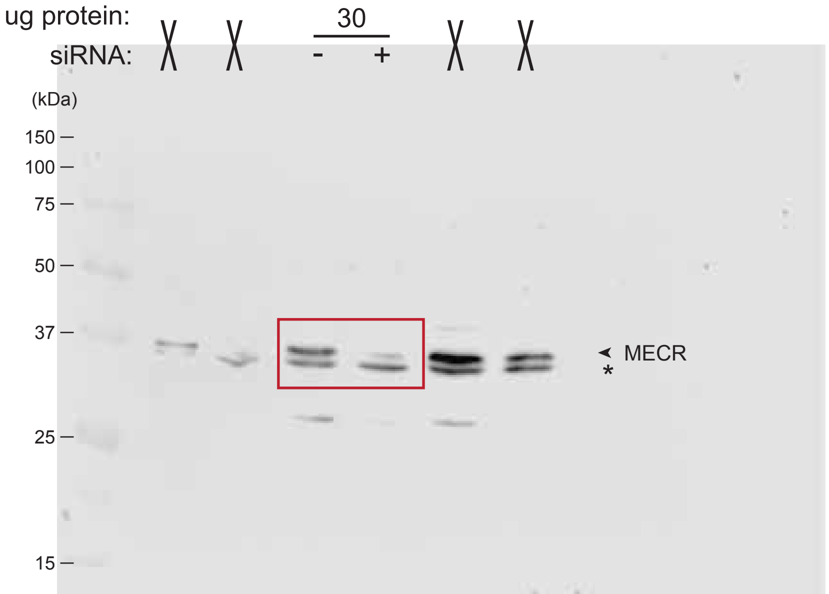

Fig. 3a (top)

IP:FLAG, Western blot, α-UL1, chemiluminescence, Odyssey Fc

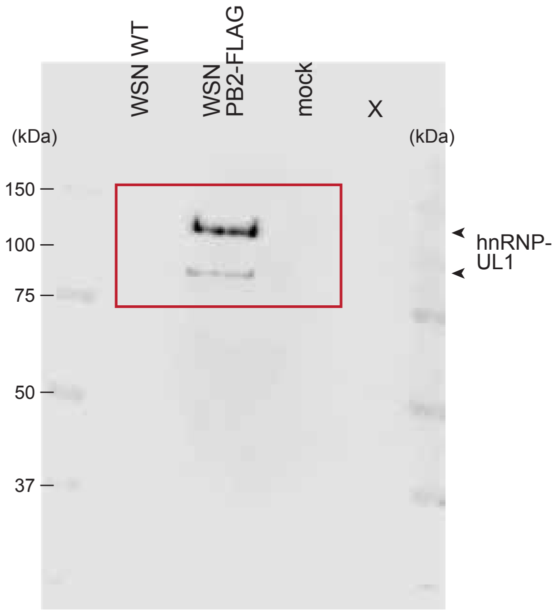

Fig. 3a (top)

IP:FLAG, Western blot, α-PB1, chemiluminescence, Odyssey Fc

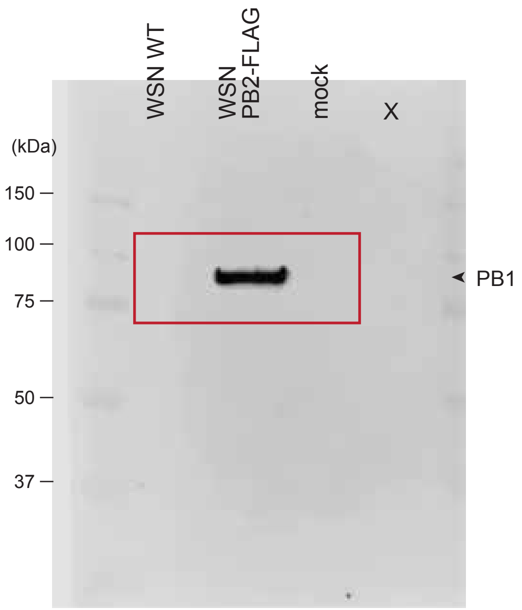

Fig. 3a (bottom)

input, Western blot, α-UL1, chemiluminescence, Odyssey Fc

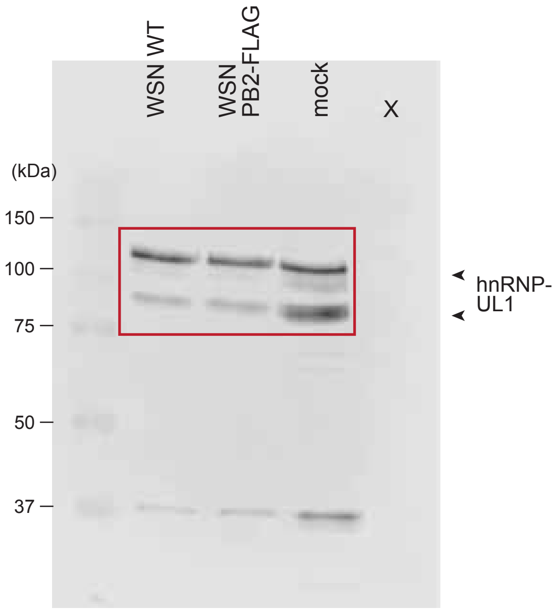

Fig. 3a (bottom)

input, Western blot, α-PB1/FLAG, chemiluminescence, Odyssey Fc

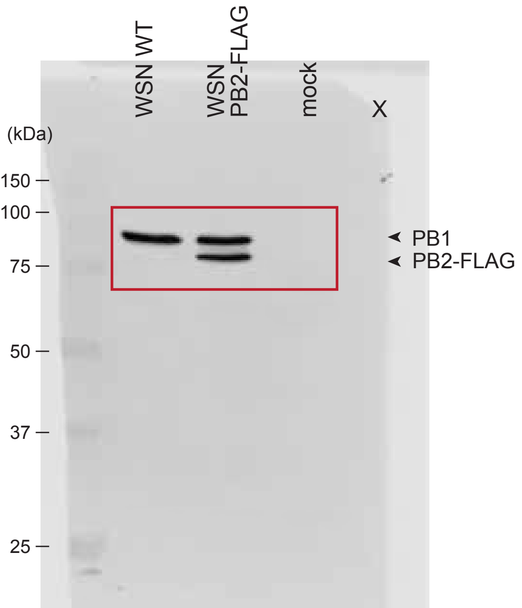

Fig. 3c (top)

IP:IgG/V5, Western blot, α-FLAG, chemiluminescence, Odyssey Fc

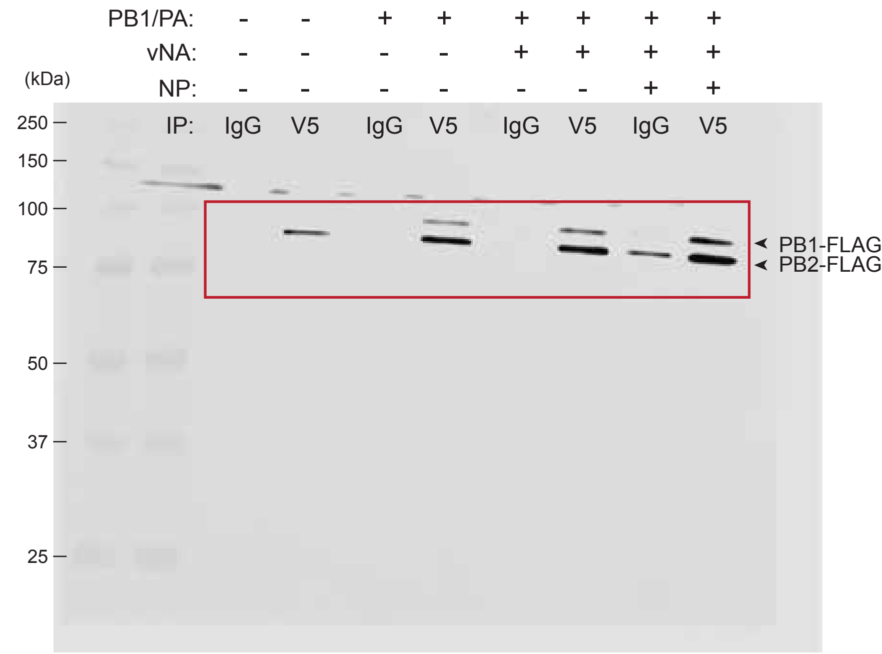

Fig. 3c (top)

IP:IgG/V5, Western blot, α-RNP, chemiluminescence, Odyssey Fc

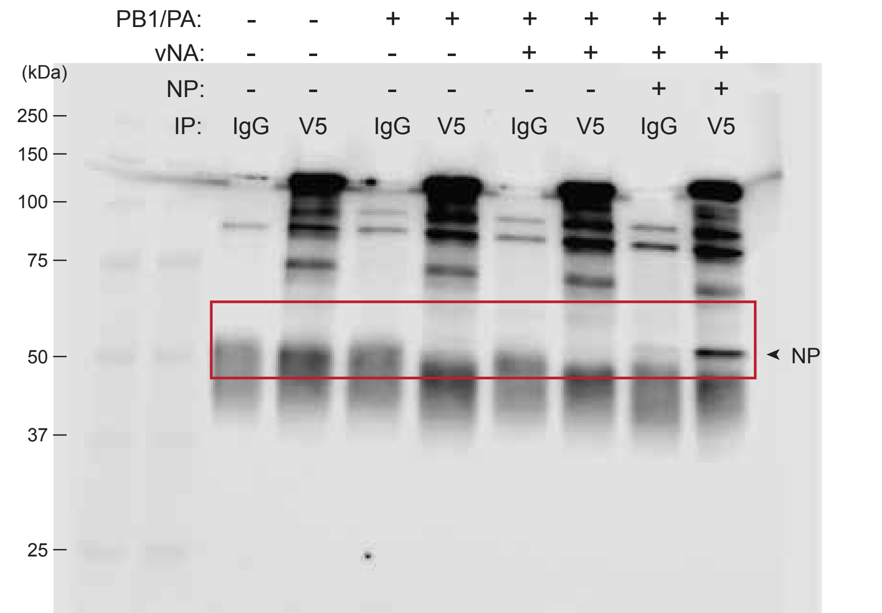

Fig. 3c (top)

IP:IgG/V5, Western blot, α-V5, chemiluminescence, Odyssey Fc

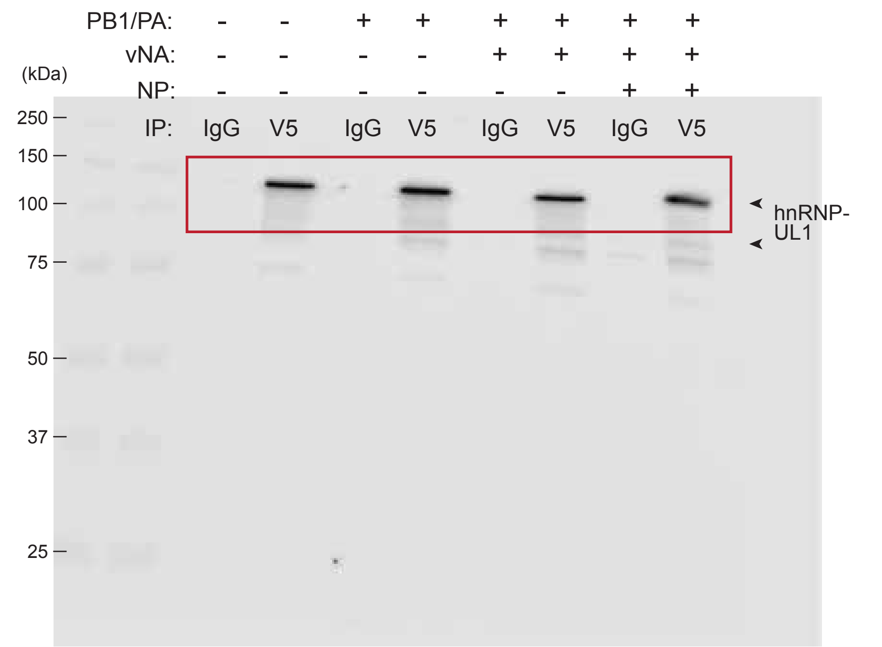

Fig. 3c (bottom)

input, Western blot, α-FLAG, chemiluminescence, Odyssey Fc

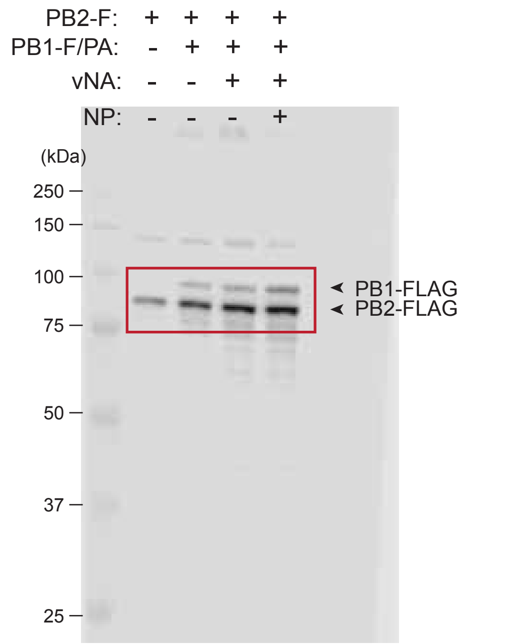

Fig. 3c (bottom)

input, Western blot, α-RNP, chemiluminescence, Odyssey Fc

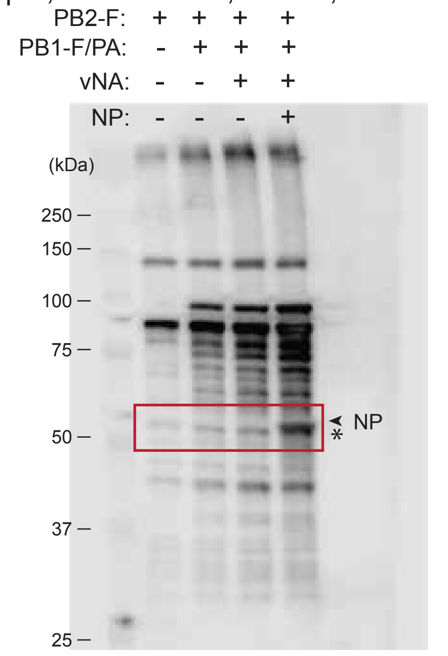

Fig. 3c (bottom)

input, Western blot, α-V5, chemiluminescence, Odyssey Fc

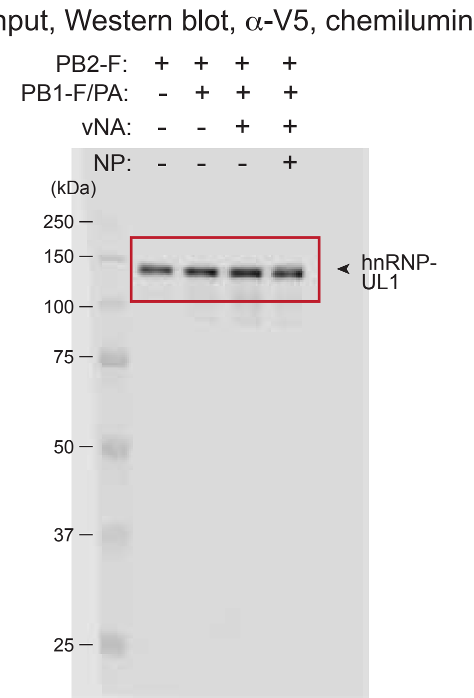

Fig. 3d (top)

IP:IgG/V5, Western blot, α-FLAG, chemiluminescence, Odyssey Fc

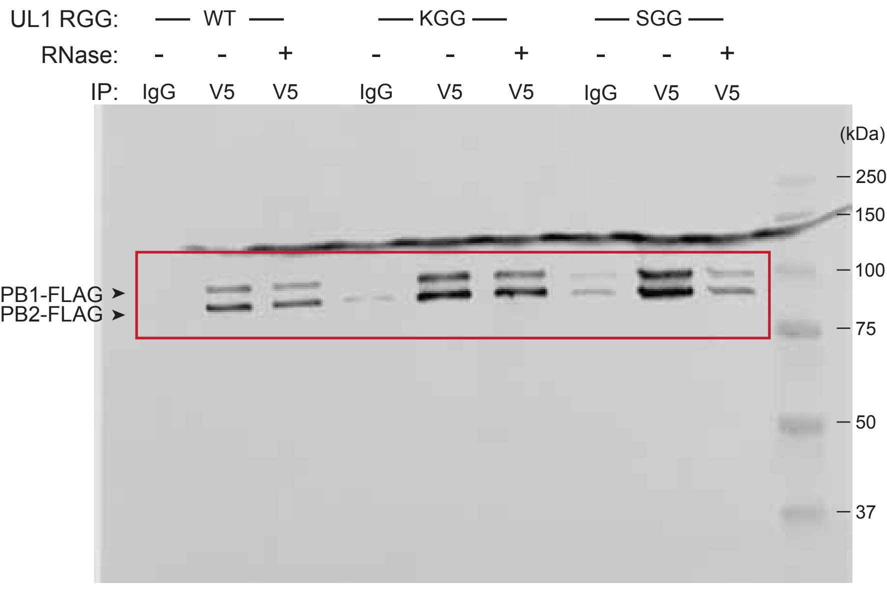

Fig. 3d (top)

IP:IgG/V5, Western blot, α-V5, chemiluminescence, Odyssey Fc

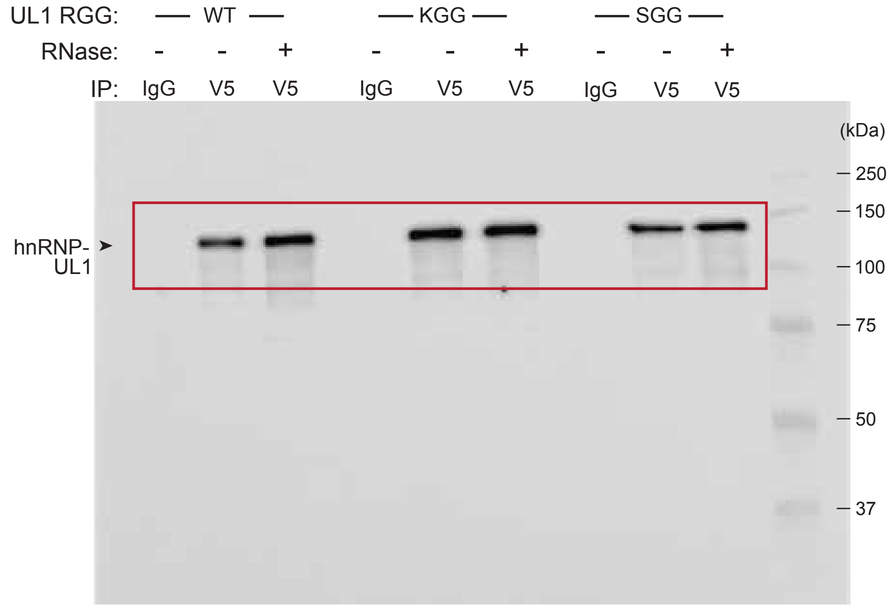

Fig. 3d (bottom)

input, Western blot, α-FLAG, chemiluminescence, Odyssey Fc

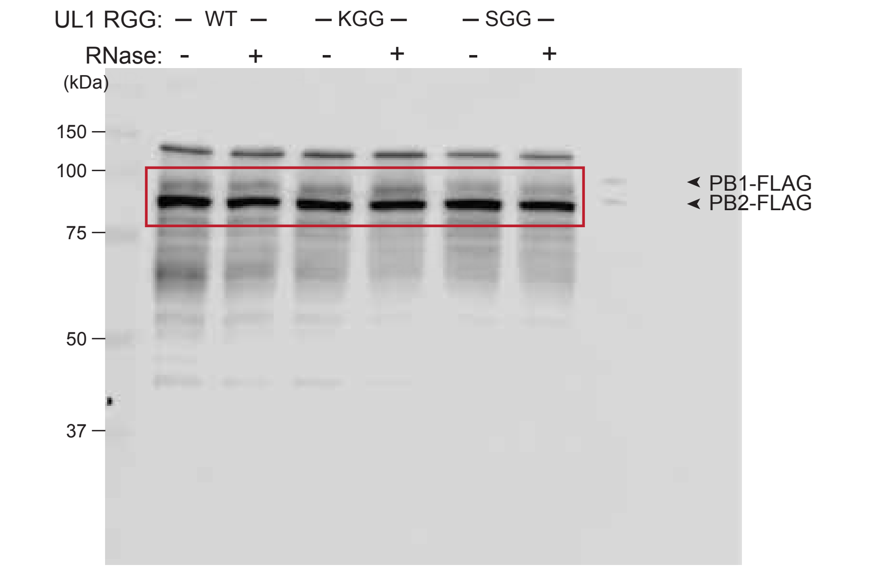

Fig. 3d (bottom)

input, Western blot, α-V5, chemiluminescence, Odyssey Fc

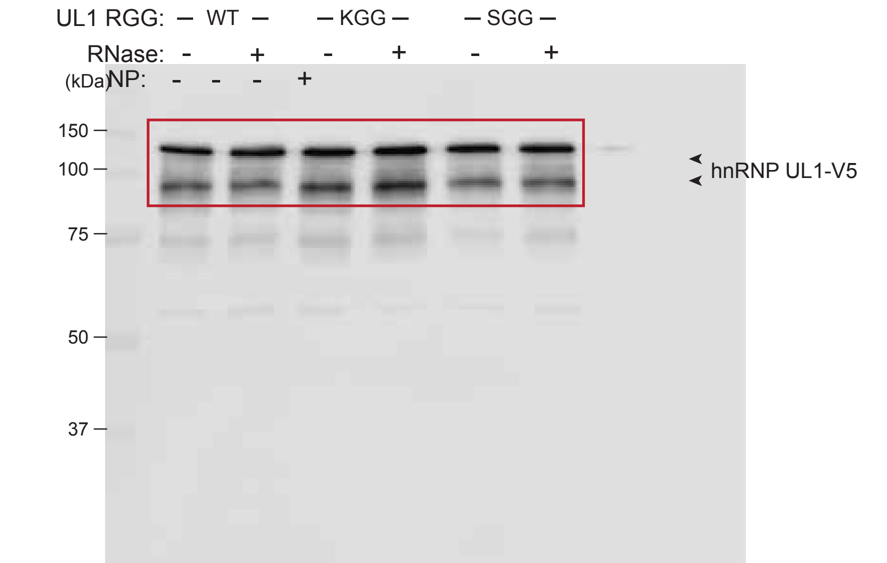

Fig. 4d (top)

Lipoic acid, Western blot, α-LA, chemiluminescence, Odyssey Fc

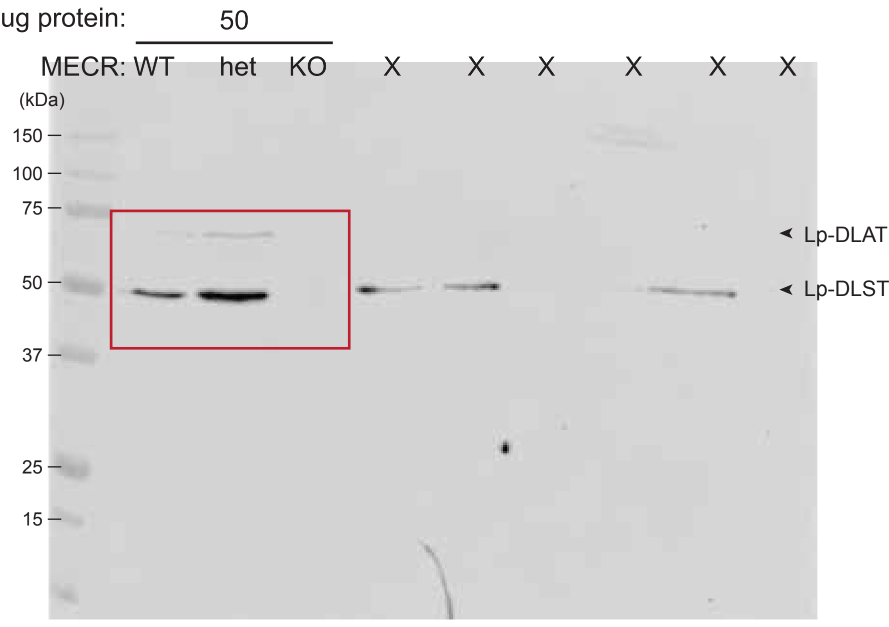

Fig. 4d (middle)

MECR, Western blot, α-MECR, chemiluminescence, Odyssey Fc

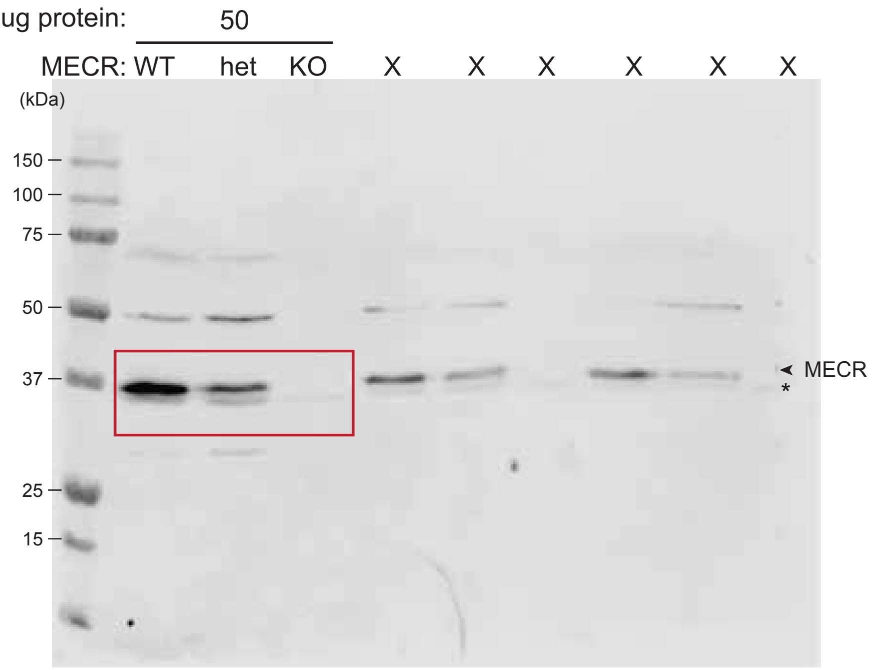

Fig. 4d (bottom)

tubulin, Western blot, α-tubulin, chemiluminescence, Odyssey Fc

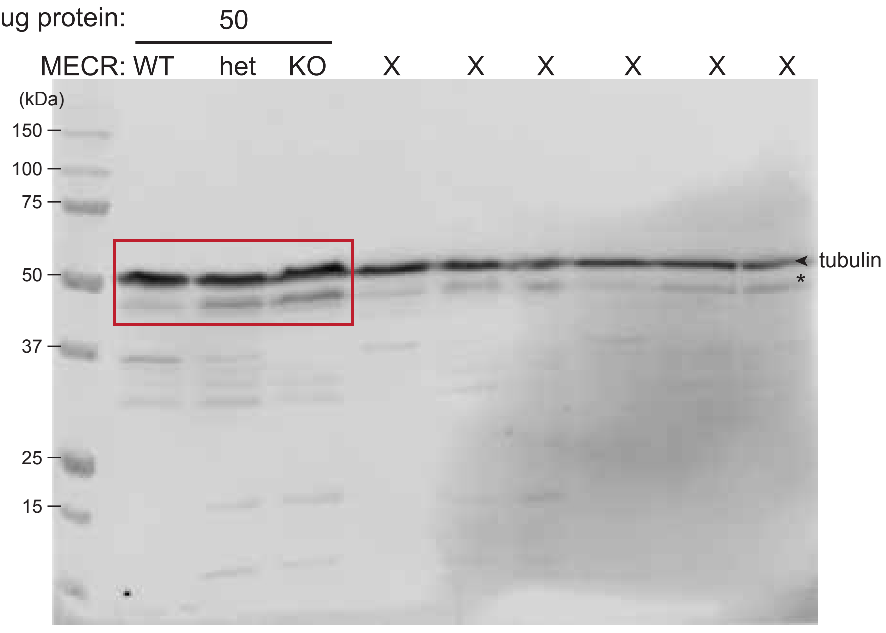

Fig. 4e (top)

MECR, Western blot, α-MECR, chemiluminescence, Odyssey Fc

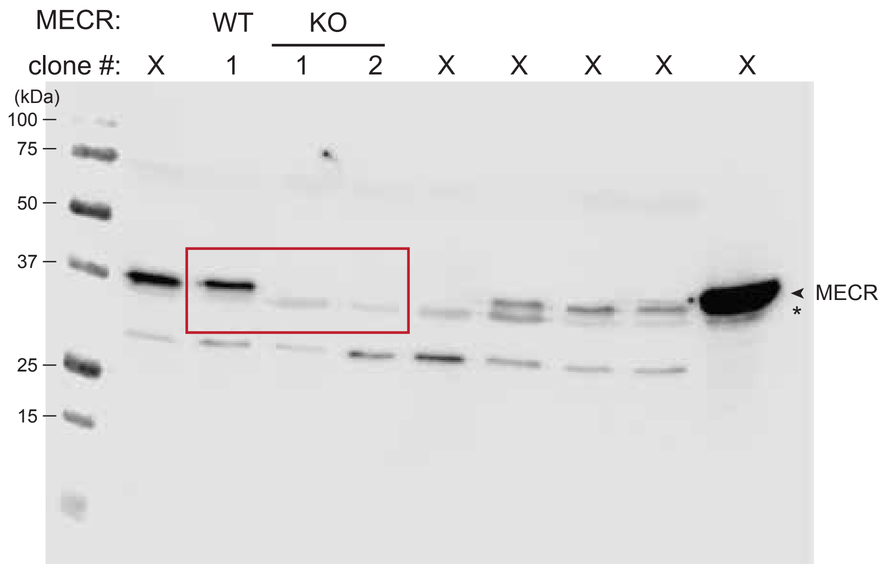

Fig. 4e (bottom)

protein, Western blot, coomassie brilliant blue (CBB), PC photoscanner

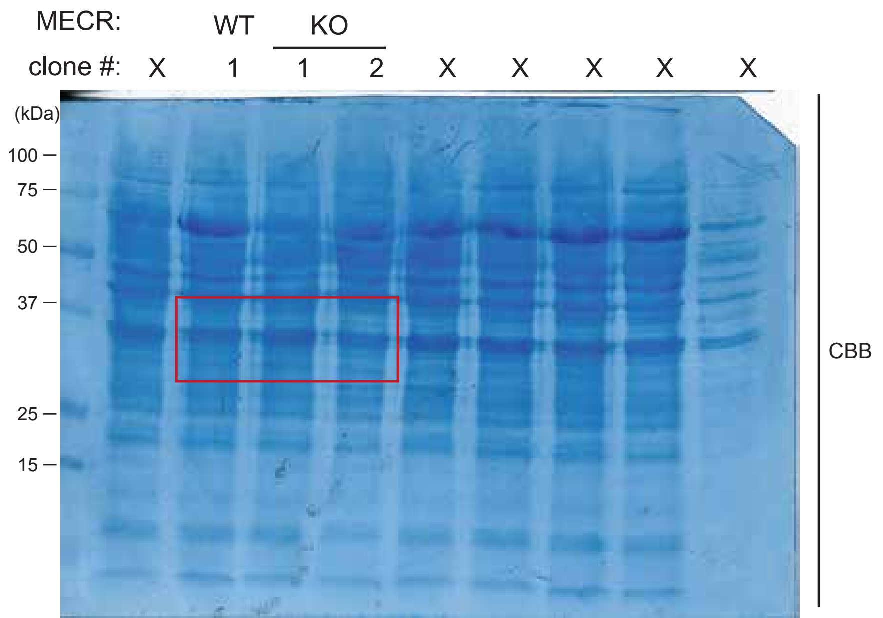

Fig. 5b (left)

cMECR-V5, Western blot, α-V5, NIR-800, Odyssey Fc

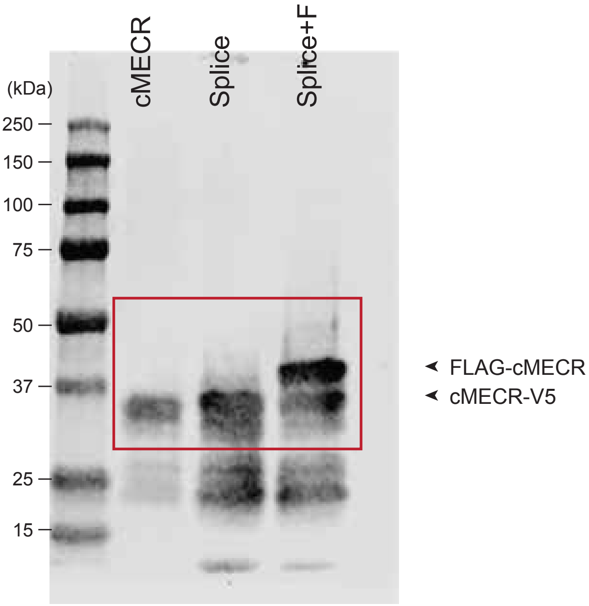

Fig. 5b (right)

cMECR-splice, Western blot, α-FLAG, NIR-700, Odyssey Fc

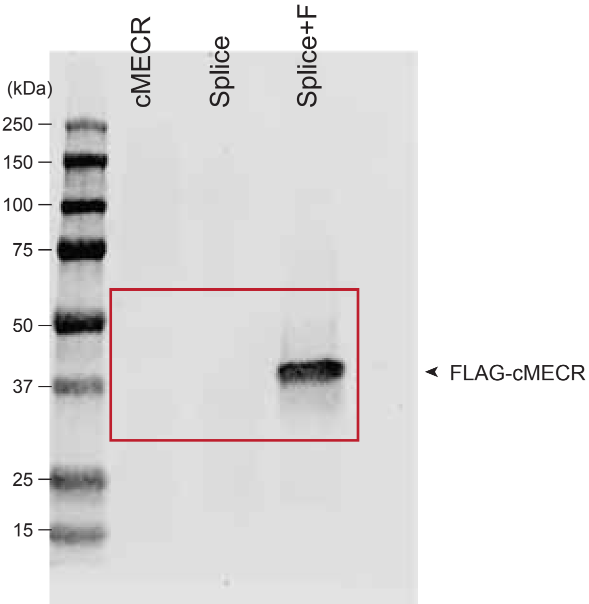

Fig. 5d (top)

IP:IgG/V5, Western blot, α-FLAG, chemiluminescence, Odyssey Fc

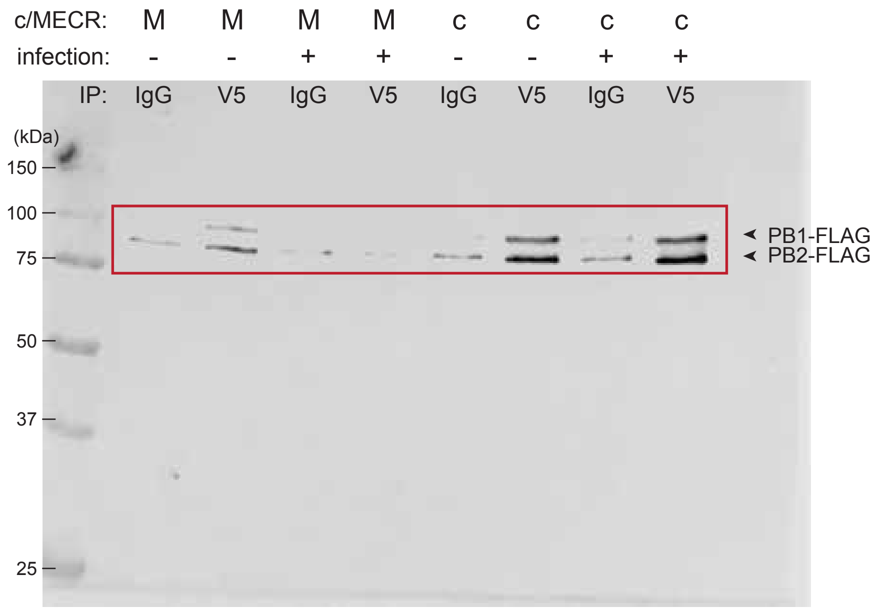

Fig. 5d (top)

IP:IgG/V5, Western blot, α-V5, chemiluminescence, Odyssey Fc

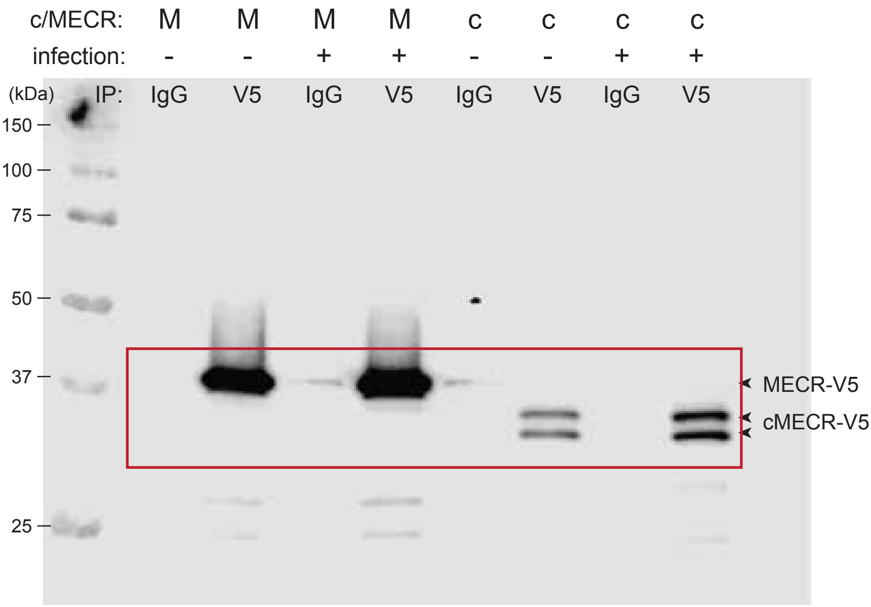

Fig. 5d (bottom)

input, Western blot, α-FLAG, chemiluminescence, Odyssey Fc

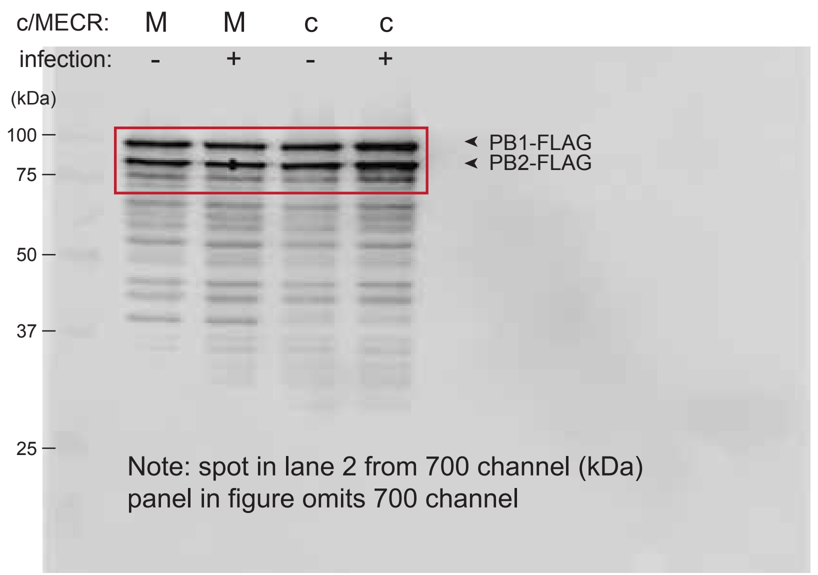

Fig. 5d (bottom)

input, Western blot, α-V5, chemiluminescence, Odyssey Fc

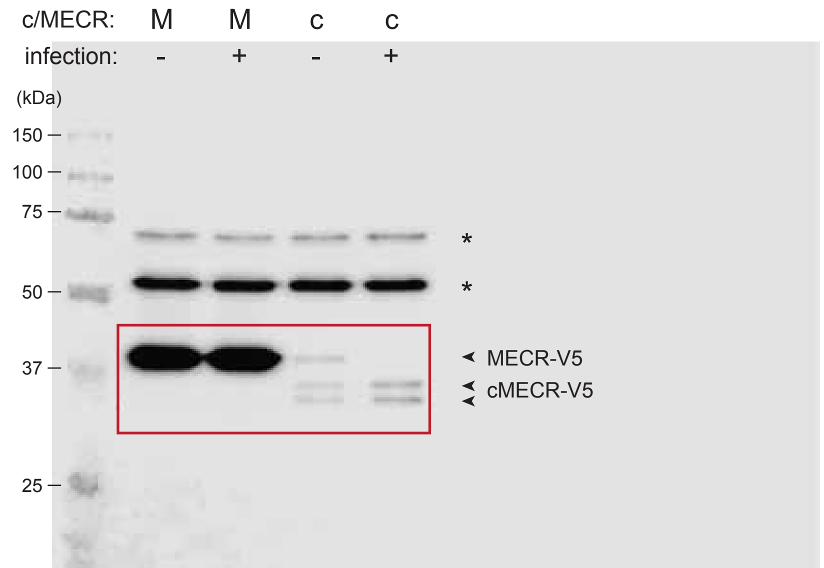

Fig. 5e (top)

IP:mCh, Western blot, α-FLAG, chemiluminescence, Odyssey Fc

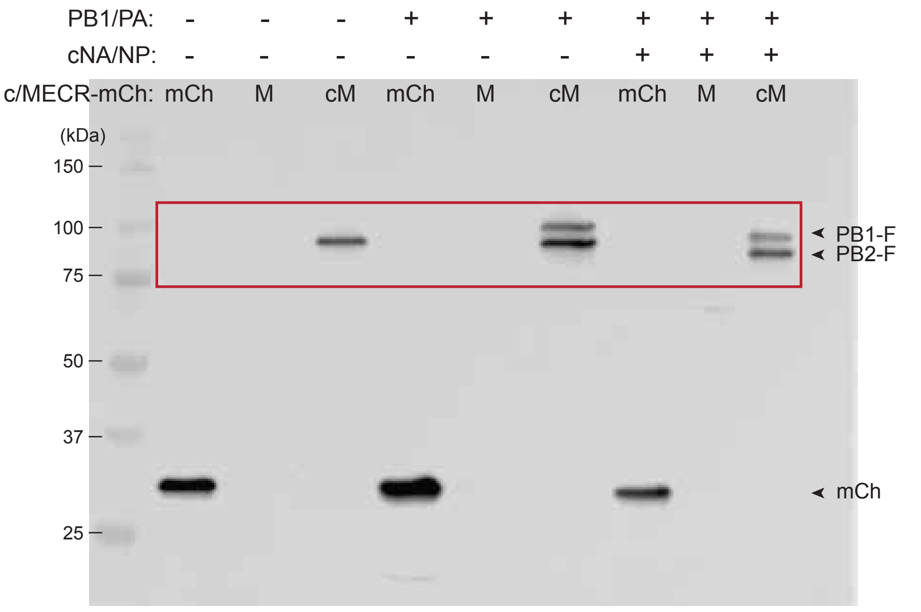

Fig. 5e (top)

IP:mCh, Western blot, α-RNP, chemiluminescence, Odyssey Fc

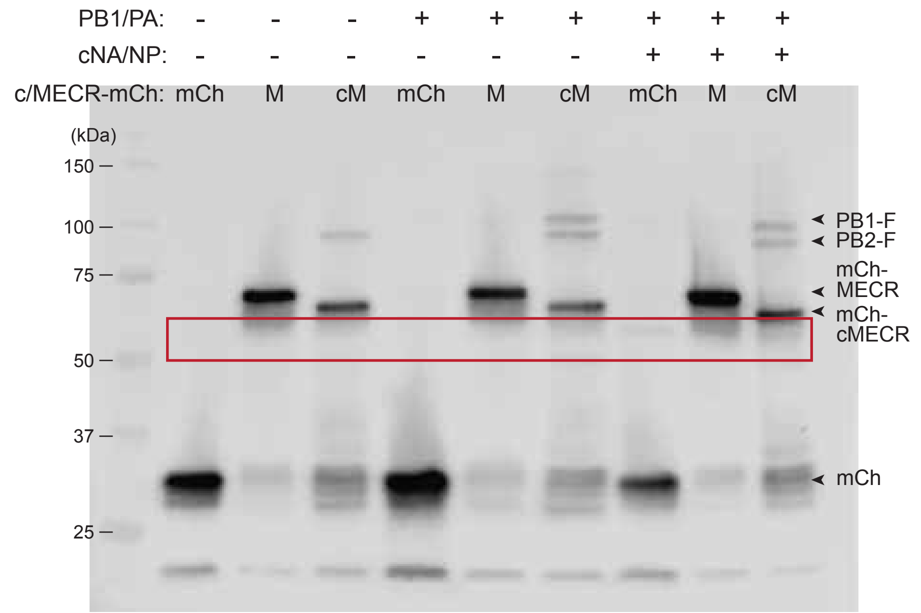

Fig. 5e (top)

IP:mCh, Western blot, α-RFP, chemiluminescence, Odyssey Fc

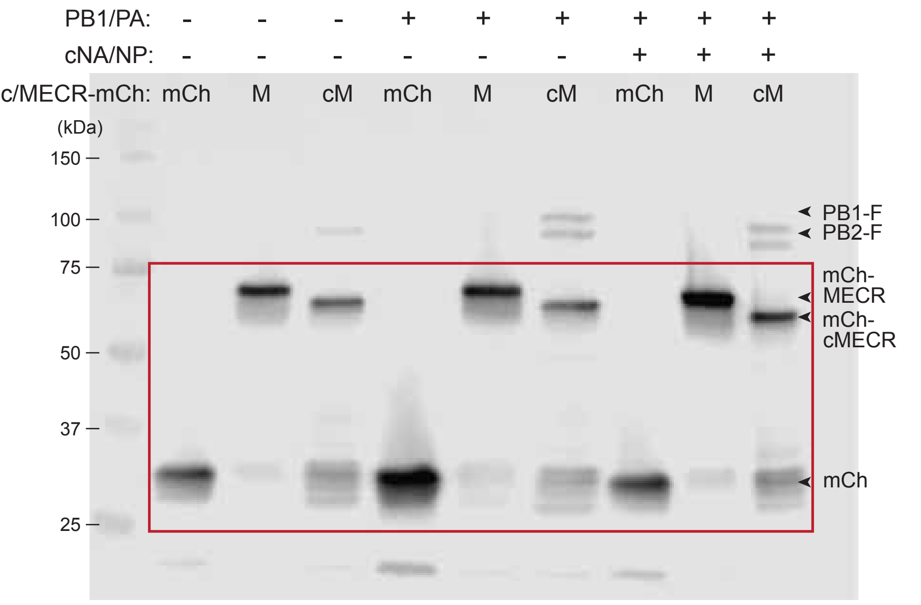

Fig. 5e (bottom)

input, Western blot, α-FLAG, chemiluminescence, Odyssey Fc

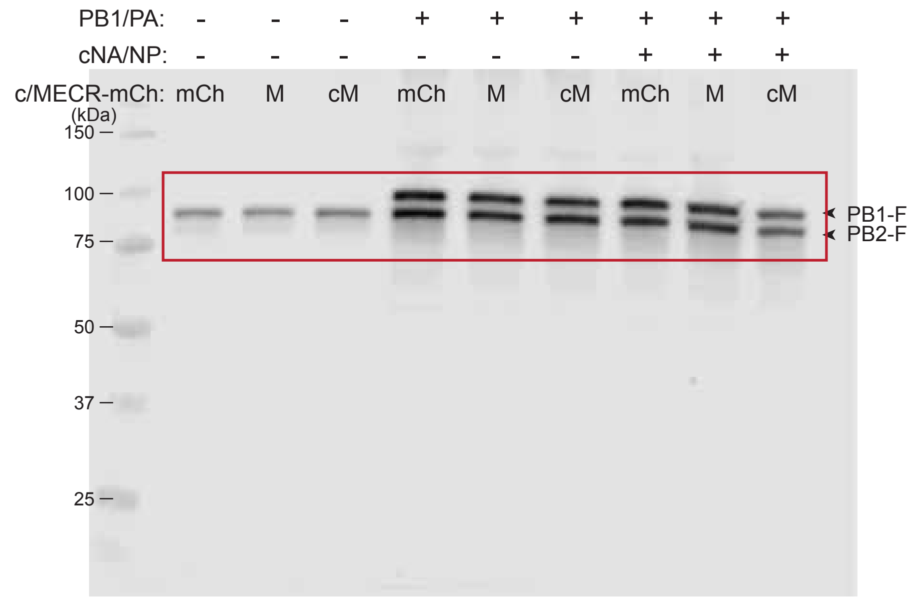

Fig. 5e (bottom)

input, Western blot, α-RNP, chemiluminescence, Odyssey Fc

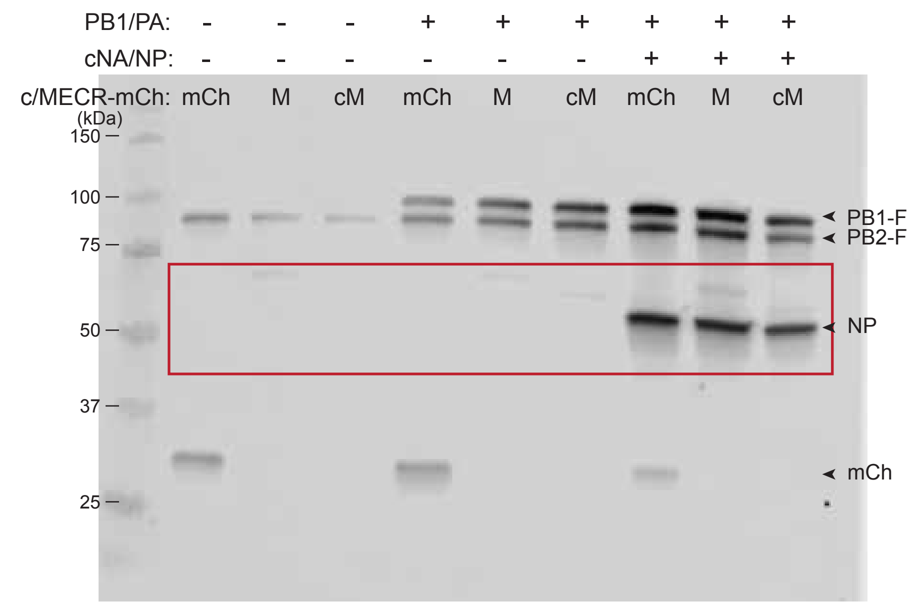

Fig. 5e (bottom)

input, Western blot, α-RFP, chemiluminescence, Odyssey Fc

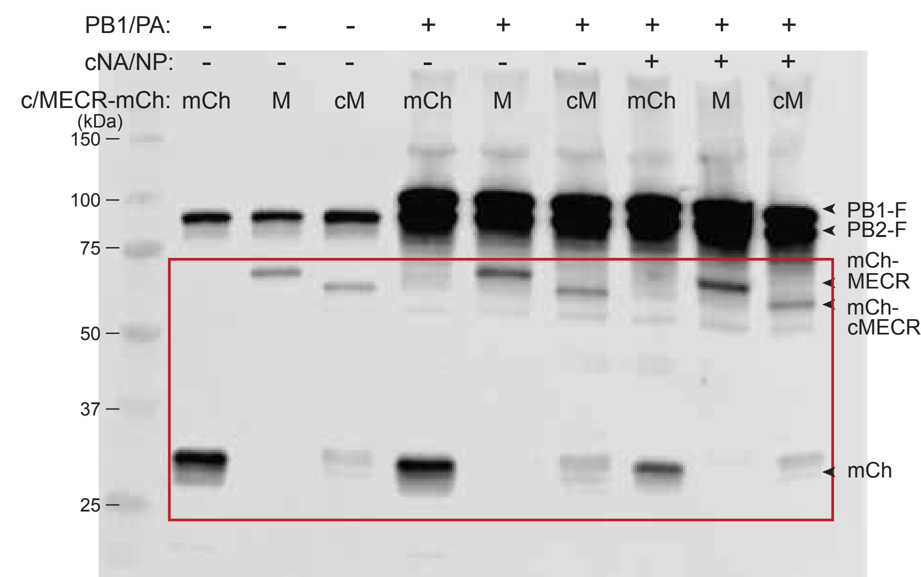

Fig. 6b (top left)
IP:mCh, Western blot, α-FLAG, chemiluminescence, Odyssey Fc

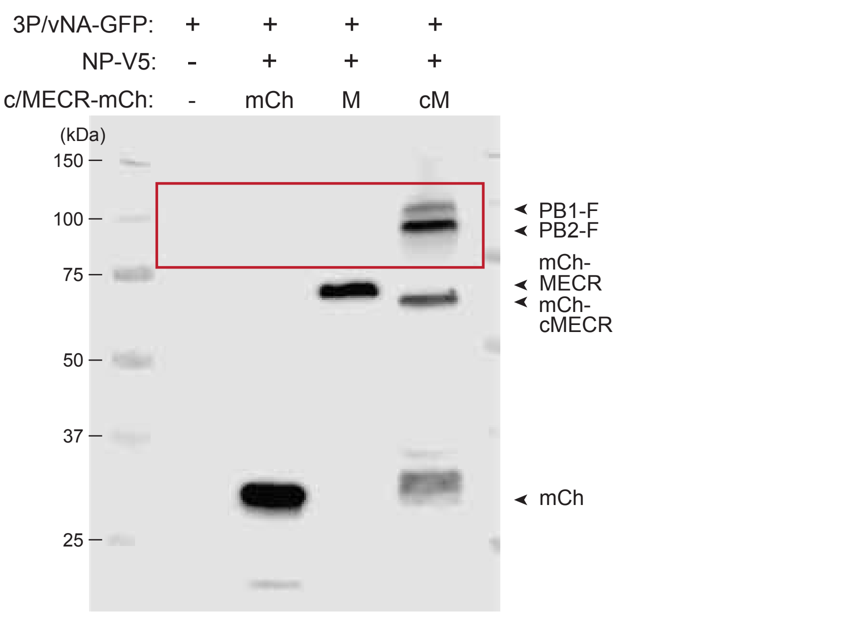

Fig. 6b (top left)
IP:mCh, Western blot, α-V5, chemiluminescence, Odyssey Fc

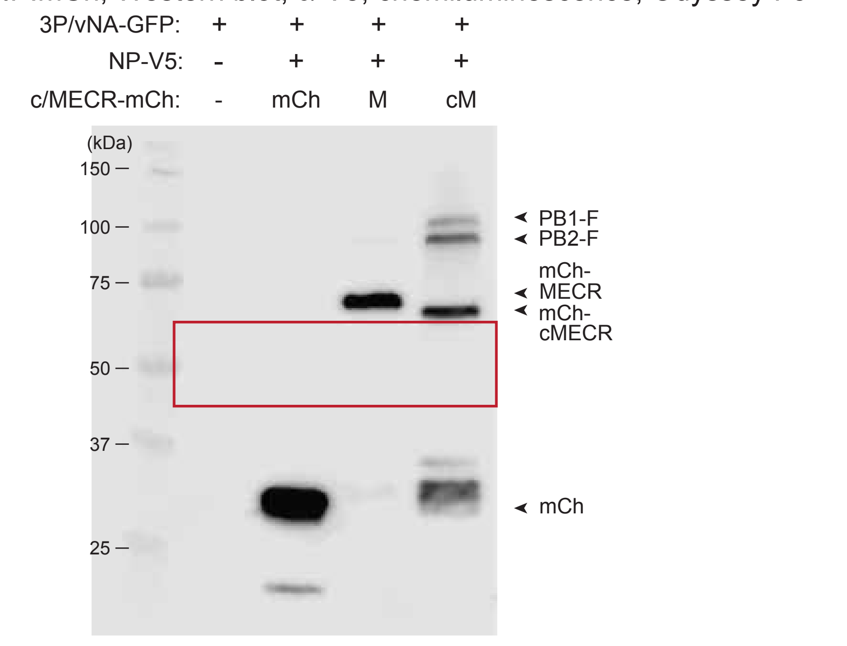

Fig. 6b (top left)
IP:mCh, Western blot, α-RFP, chemiluminescence, Odyssey Fc

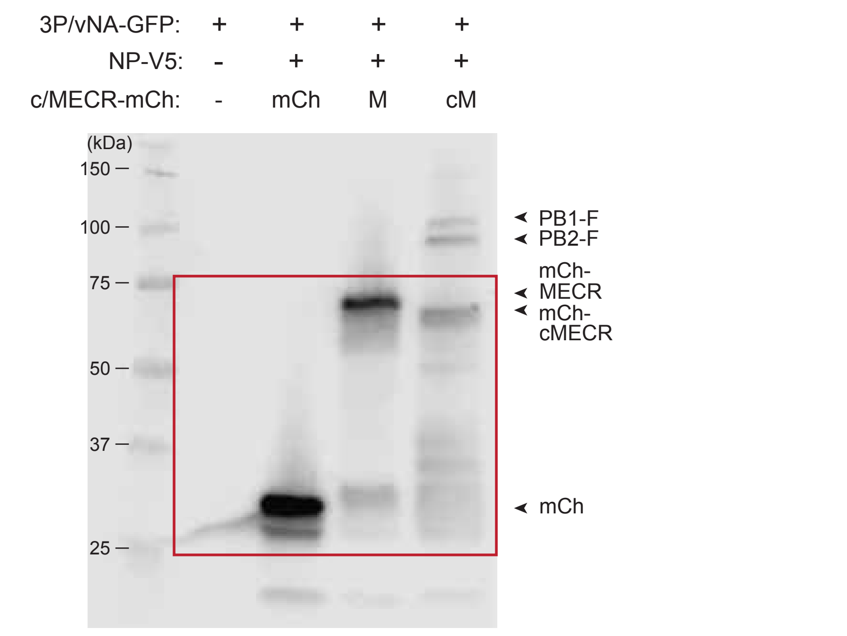

Fig. 6b (bottom left)
IP:V5, Western blot, α-FLAG, chemiluminescence, Odyssey Fc

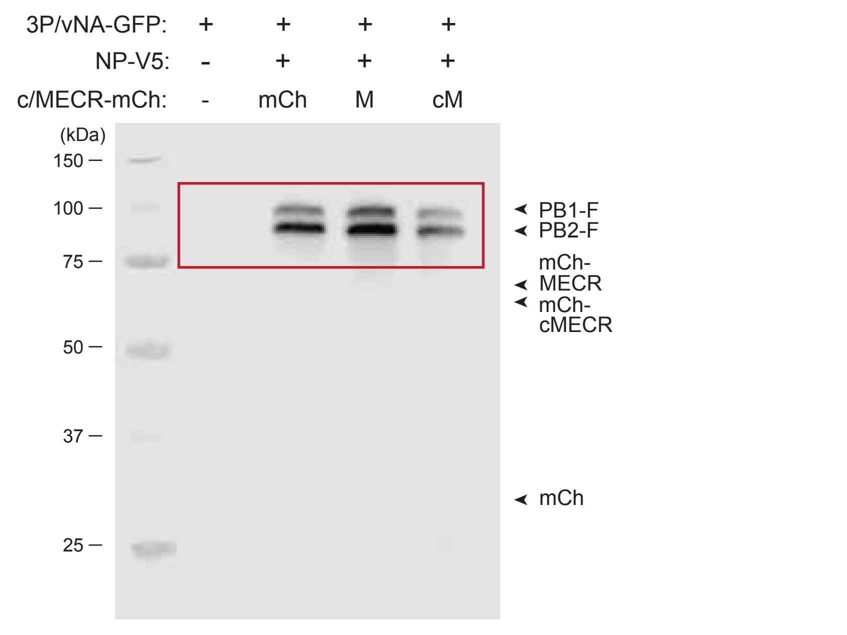

Fig. 6b (bottom left)
IP:V5, Western blot, α-V5, chemiluminescence, Odyssey Fc

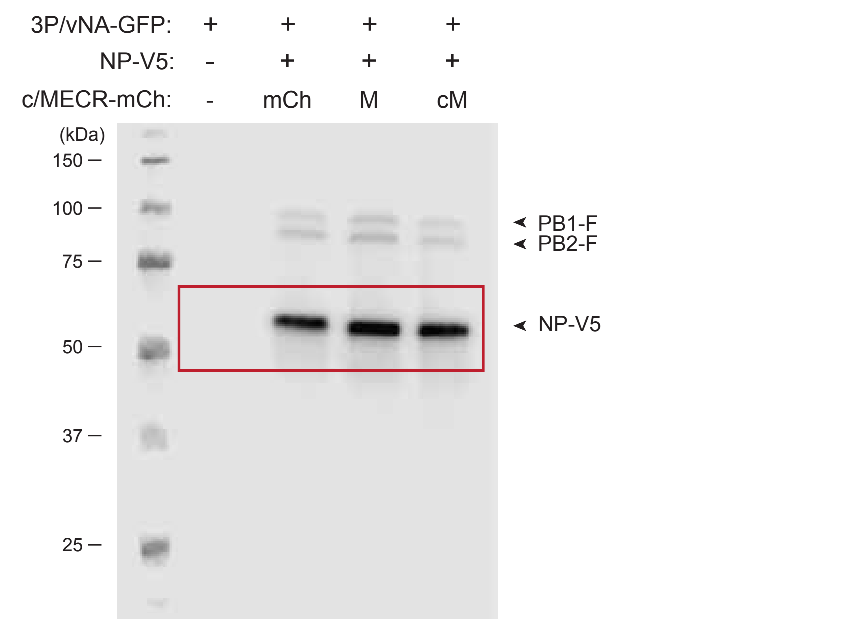

Fig. 6b (bottom left)
IP:V5, Western blot, α-RFP, chemiluminescence, Odyssey Fc

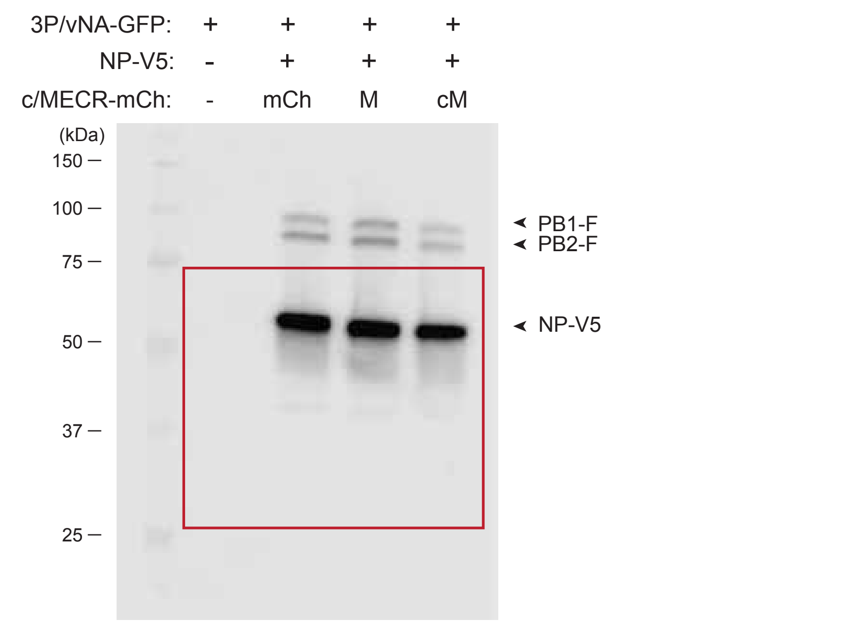

Fig. 6b (right)
input, Western blot, α-FLAG, chemiluminescence, Odyssey Fc

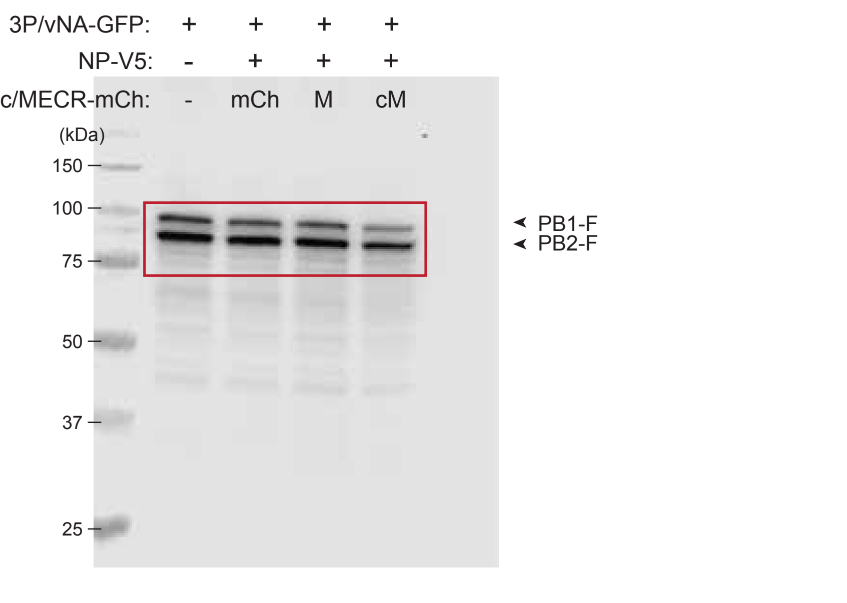

Fig. 6b (right)
input, Western blot, α-V5, chemiluminescence, Odyssey Fc

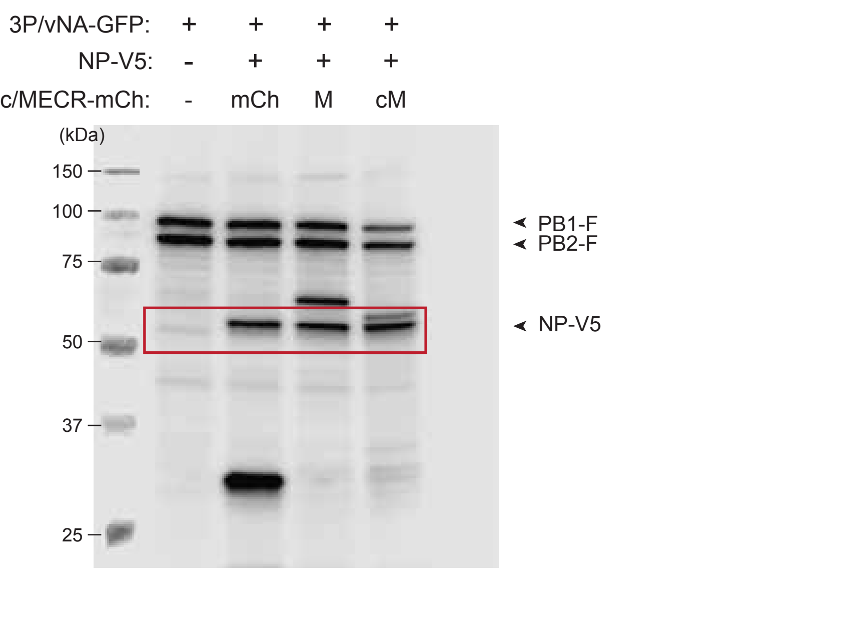

Fig. 6b (right)
input, Western blot, α-RFP, chemiluminescence, Odyssey Fc

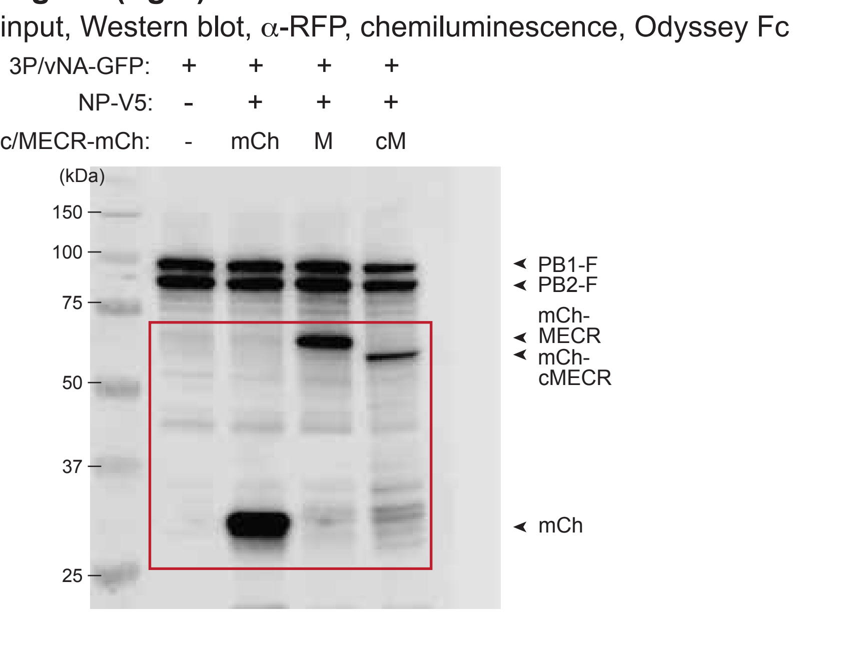

Fig. 6b (right)
input, Western blot, α-GFP, chemiluminescence, Odyssey Fc

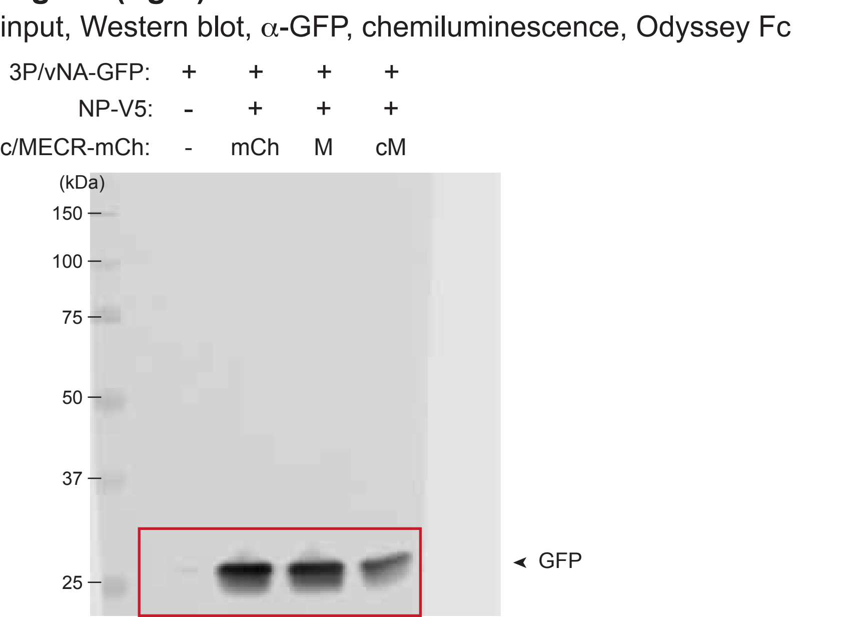

Fig. 6c (top)

IP:FLAG, Western blot, α-PB1, chemiluminescence, Odyssey Fc

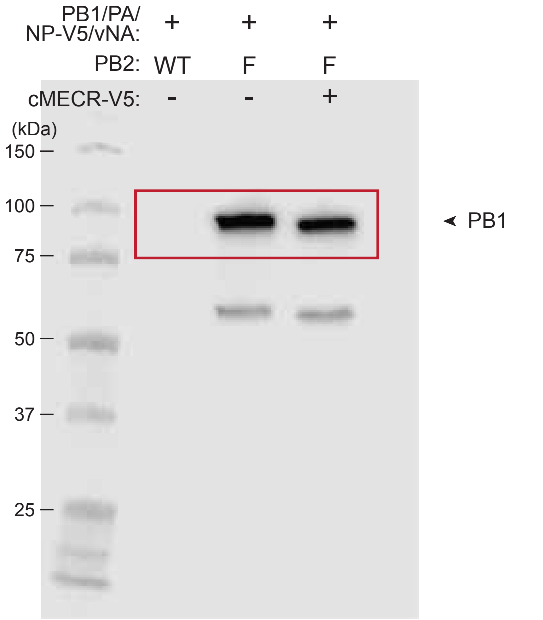

Fig. 6c (top)

IP:FLAG, Western blot, α-V5, chemiluminescence, Odyssey Fc

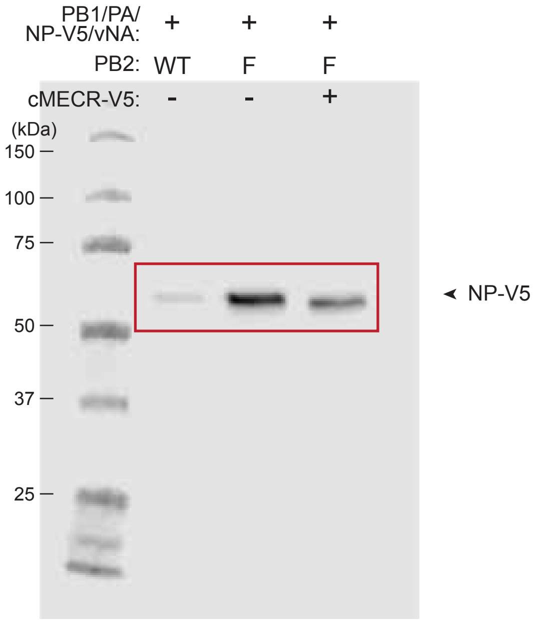

Fig. 6c (bottom)

input, Western blot, α-PB1/PB2, chemiluminescence, Odyssey Fc

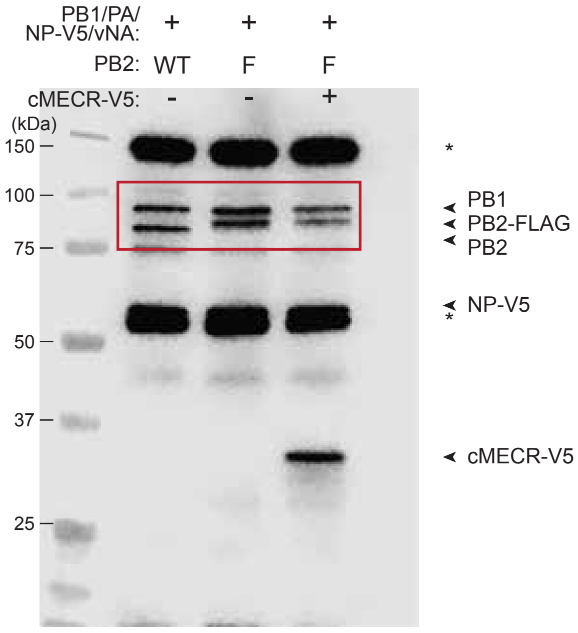

Fig. 6c (bottom)

input, Western blot, α-V5, chemiluminescence, Odyssey Fc

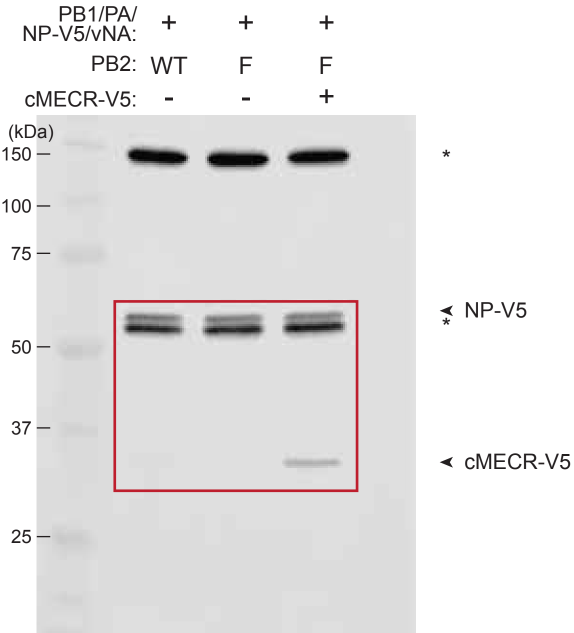

Fig. 6f (#1 from top)

c/MECR, Western blot, α-MECR, chemiluminescence, Odyssey Fc

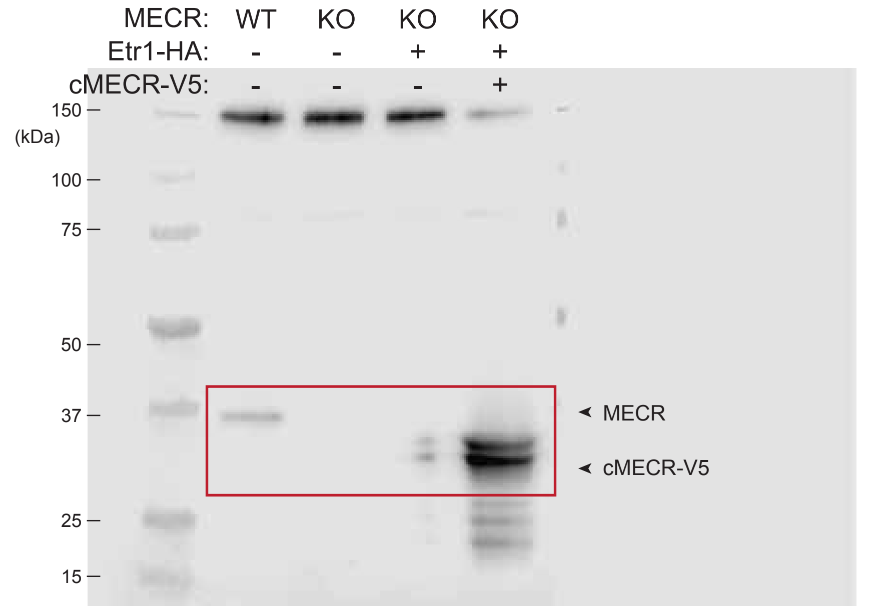

Fig. 6f (#2 from top)

Etr1, Western blot, α-HA, chemiluminescence, Odyssey Fc

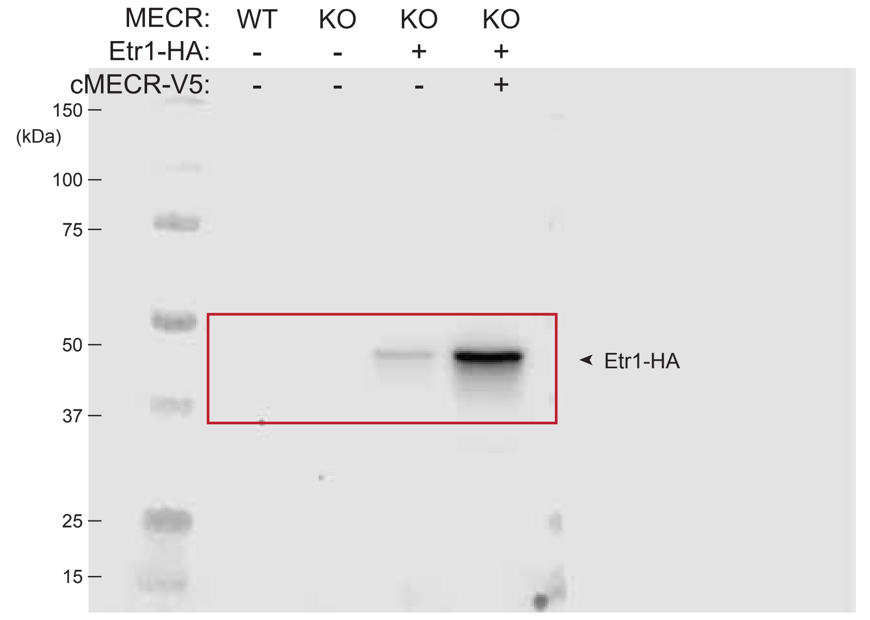

Fig. 6f (#3 from top)

Lipoic acid, Western blot, α-LA, chemiluminescence, Odyssey Fc

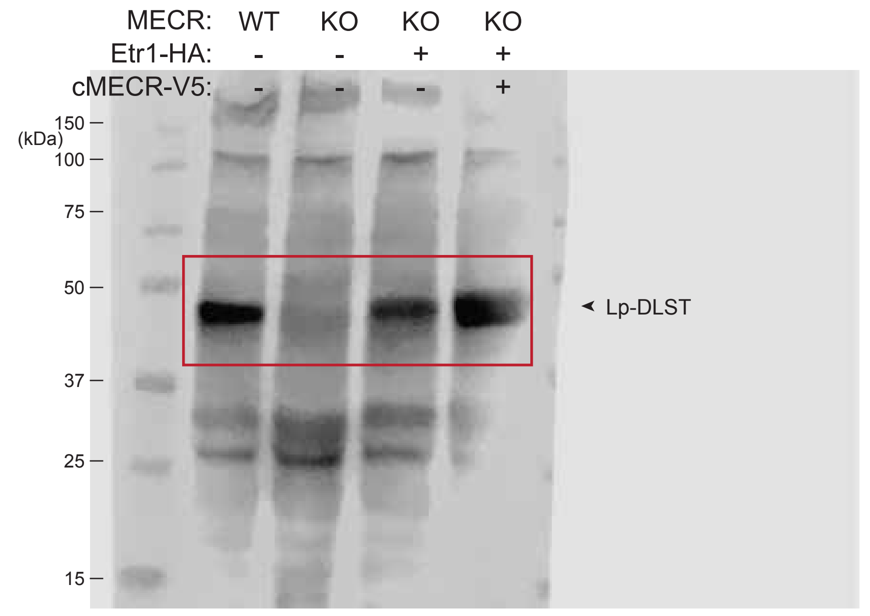

Fig. 6f (#4 from top)

cMECR, Western blot, α-V5, chemiluminescence, Odyssey Fc

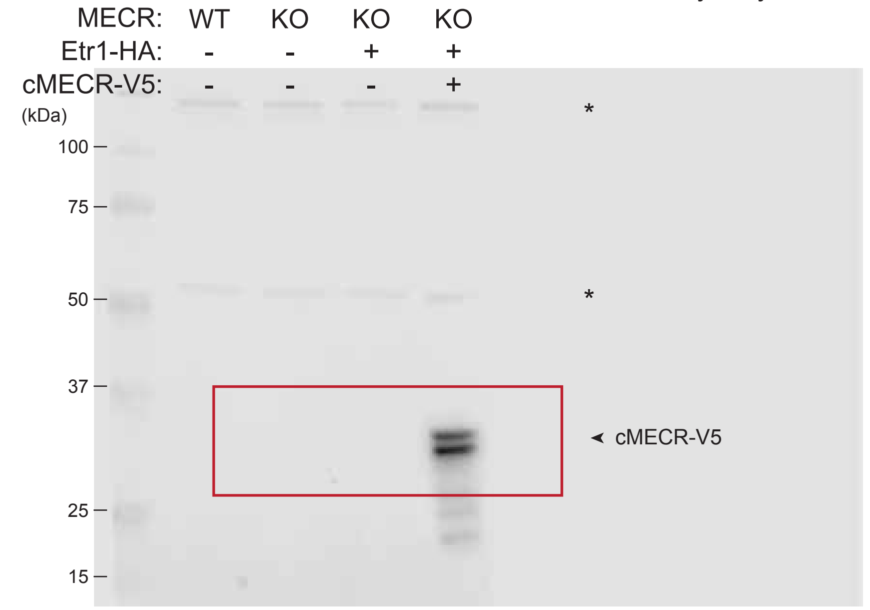

Fig. 6f (#5 from top)

protein, Western blot, coomassie brilliant blue (CBB), PC photoscanner

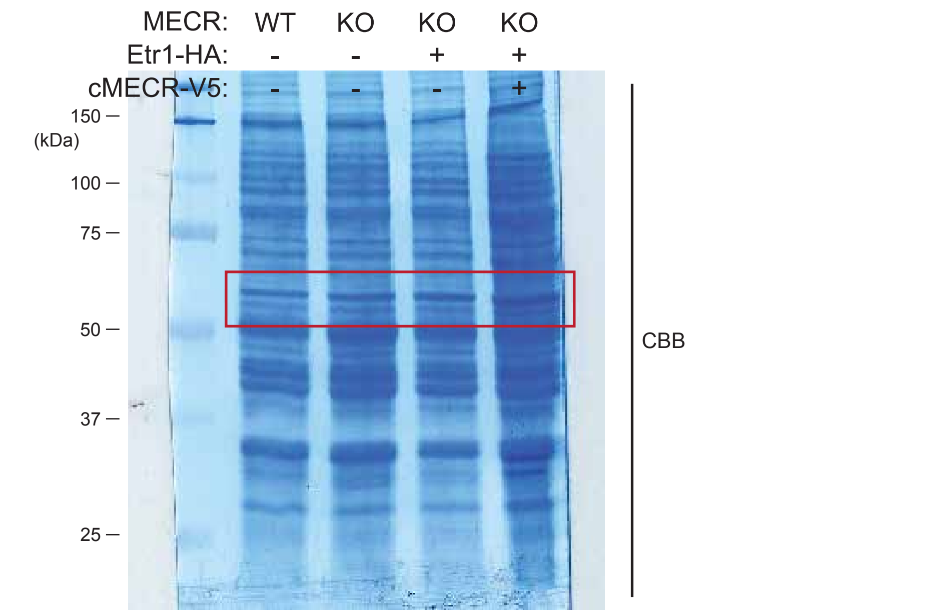

Fig. S5b (left)

MECR ex1, Agarose gel, EtBr, 600 NIR channel, Odyssey Fc

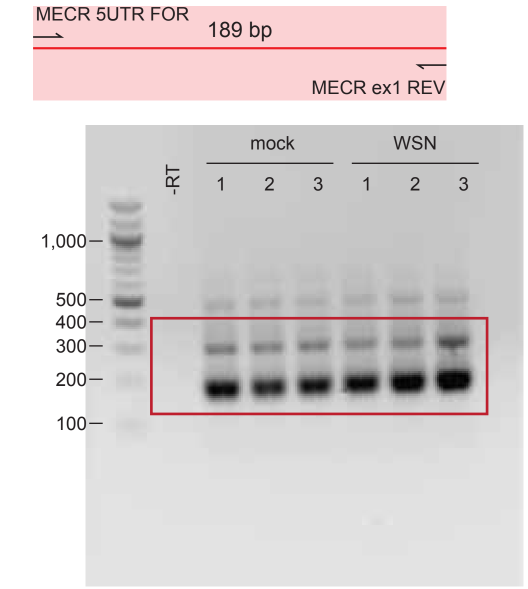

Fig. S5b (middle)

MECR ex1-2, Agarose gel, EtBr, 600 NIR channel, Odyssey Fc

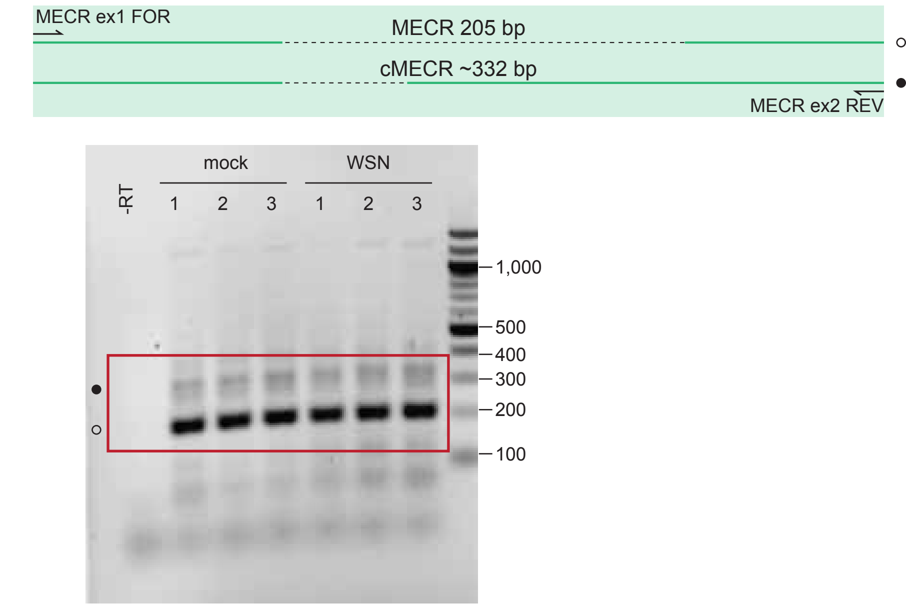

Fig. S5b (right)

MECR ex2-3, Agarose gel, EtBr, 600 NIR channel, Odyssey Fc

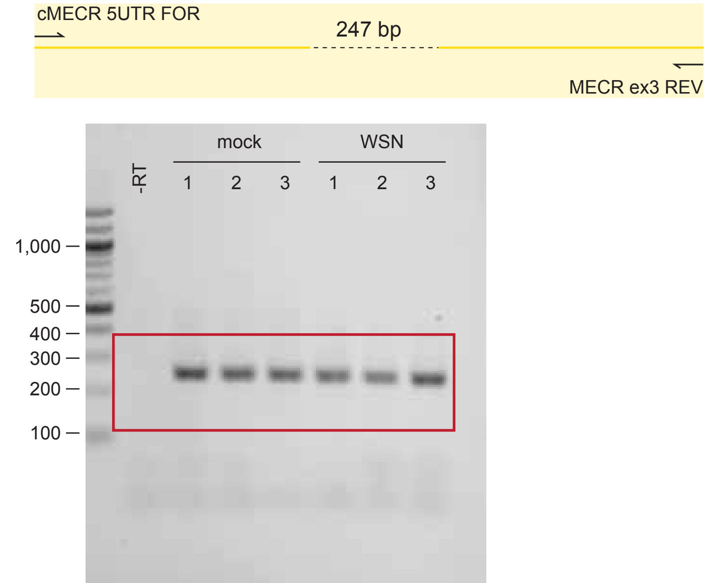

Fig. S5d (top)

IP:V5, Western blot, α-FLAG, chemiluminescence, Odyssey Fc

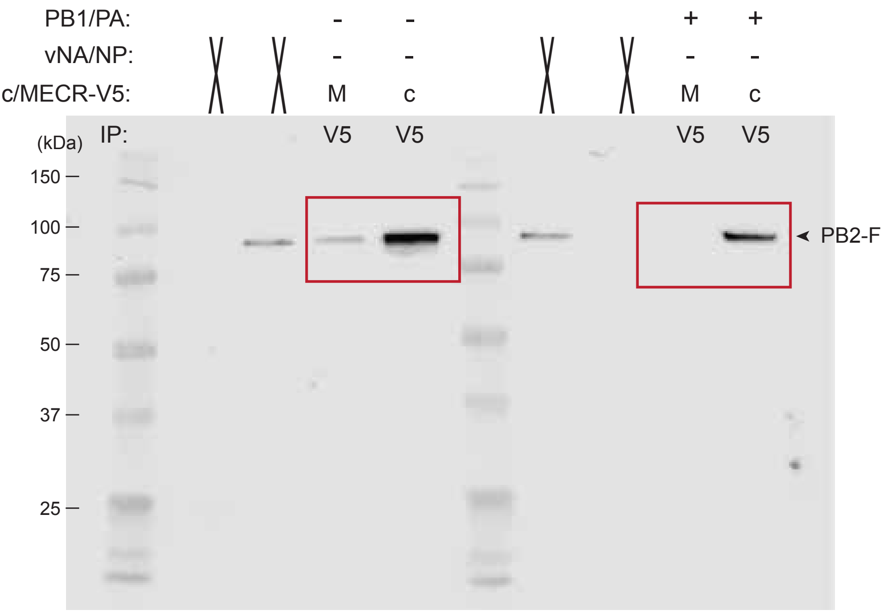

Fig. S5d (top)

IP:V5, Western blot, α-FLAG, chemiluminescence, Odyssey Fc

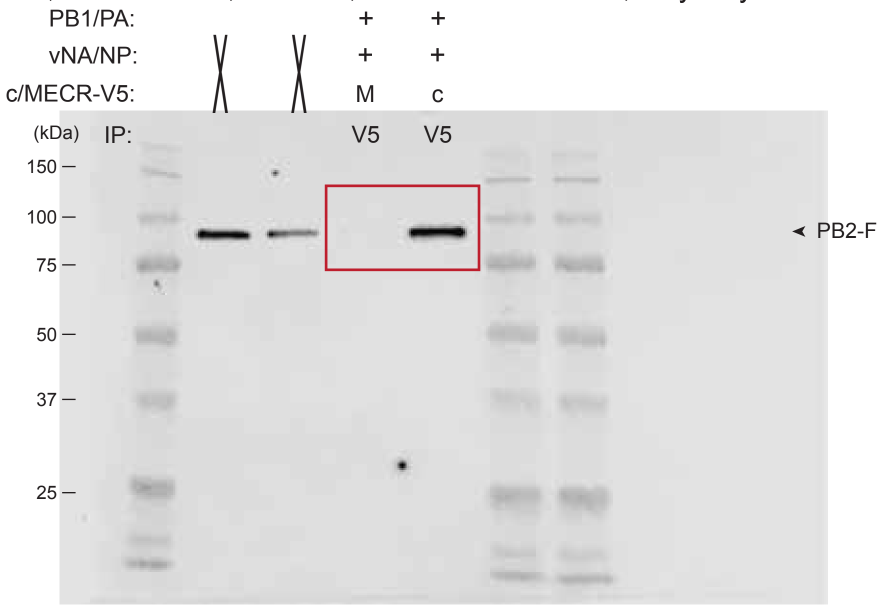

Fig. S5d (top)

IP:V5, Western blot, α-V5, chemiluminescence, Odyssey Fc

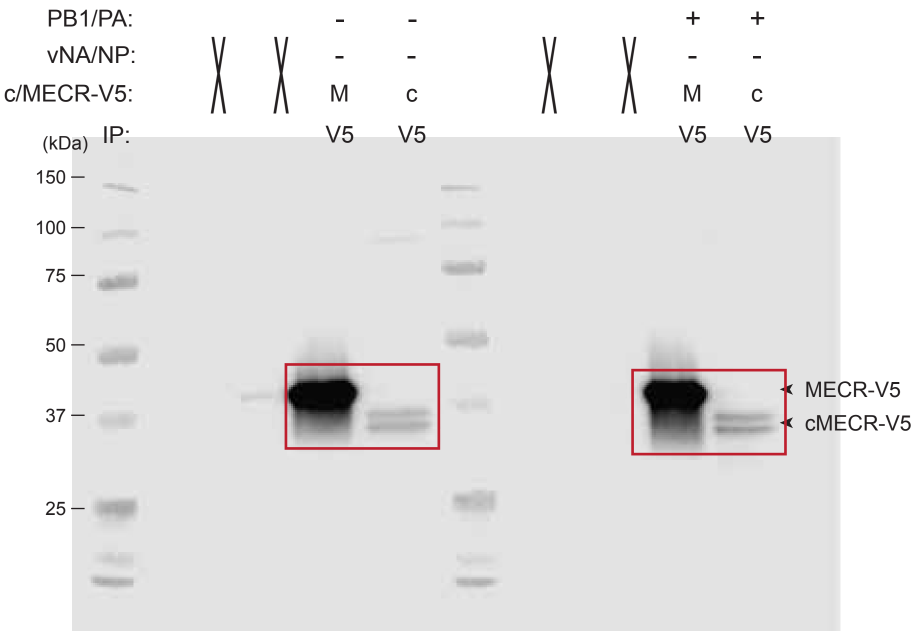

Fig. S5d (top)

IP:V5, Western blot, α-FLAG, chemiluminescence, Odyssey Fc

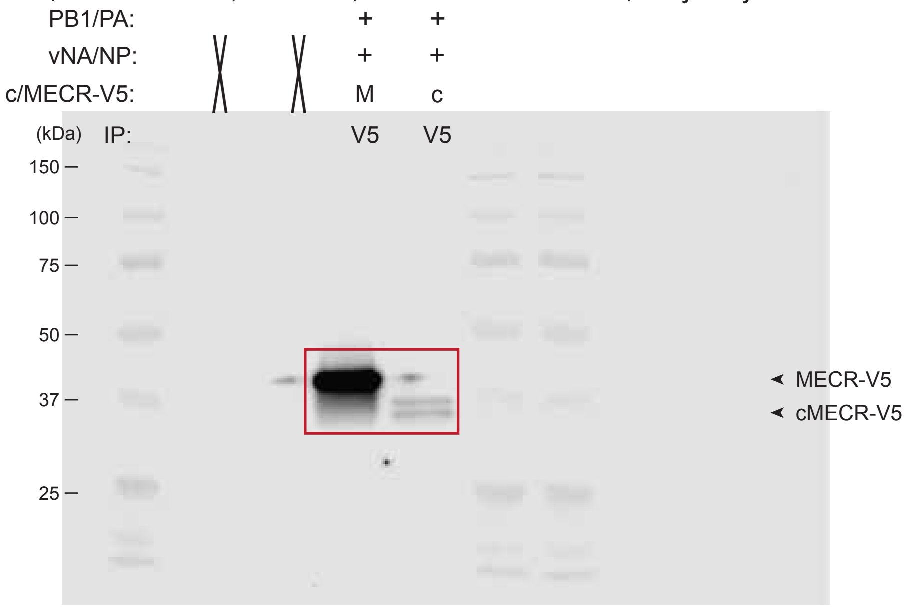

Fig. S5d (bottom)

input, Western blot, α-FLAG, chemiluminescence, Odyssey Fc

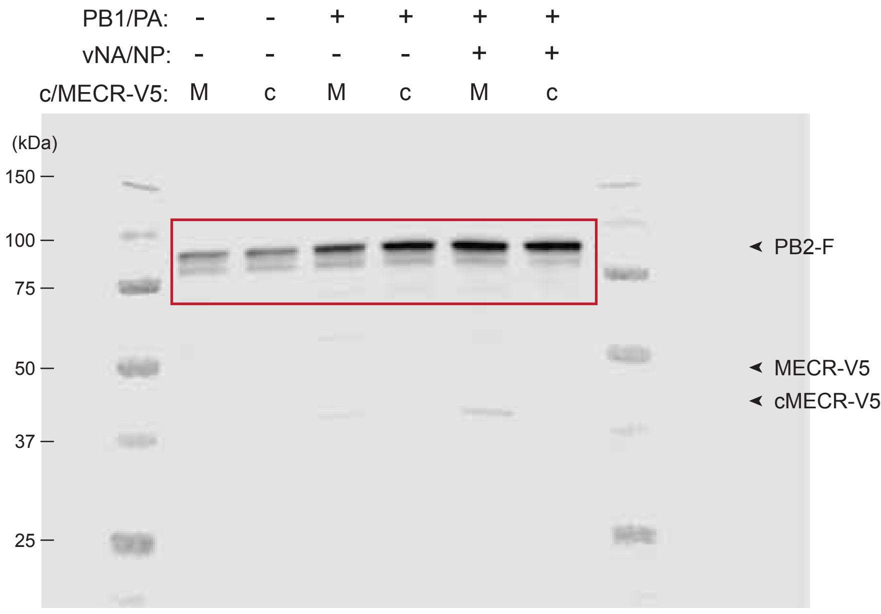

Fig. S5d (bottom)

input, Western blot, α-V5, chemiluminescence, Odyssey Fc

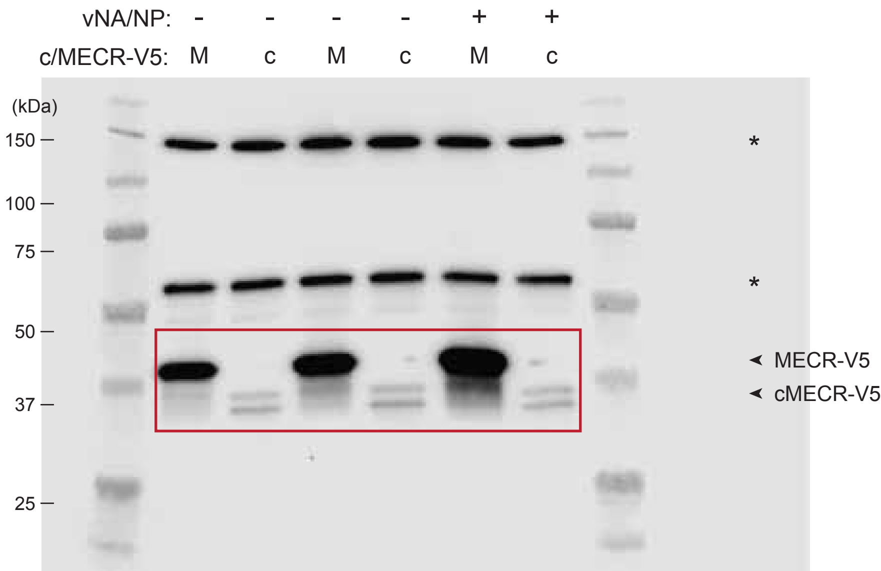

Fig. S5f (left)

IP:FLAG, Western blot, α-PB1, chemiluminescence, Odyssey Fc

PB1/PA:	+	+	X	X	+	+
cMECR-AirID:	-	-			+	+
PB2:	WT	F			WT	F

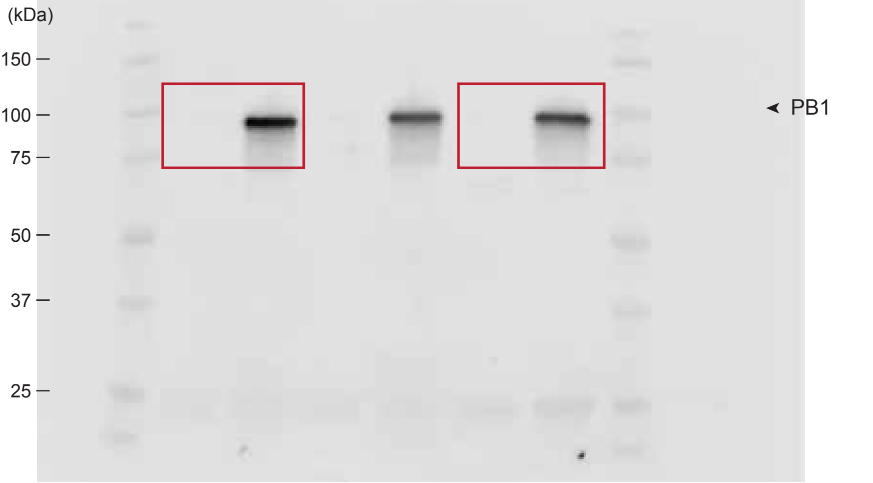

Fig. S5f (left)

IP:FLAG, Western blot, α-SA, chemiluminescence, Odyssey Fc

PB1/PA:	+	+	X	X	+	+
cMECR-AirID:	-	-			+	+
PB2:	WT	F			WT	F

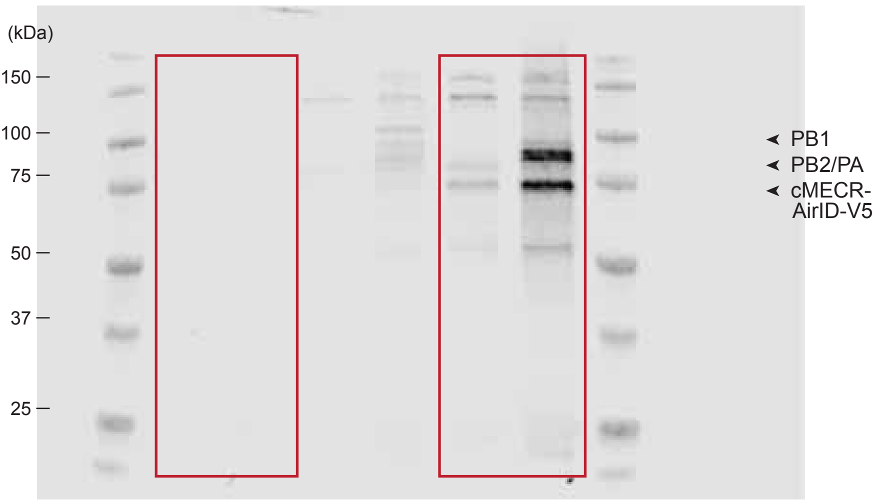

Fig. S5f (right)

input, Western blot, α-V5, chemiluminescence, Odyssey Fc

PB1/PA:	+	+	X	X	+	+
cMECR-AirID:	-	-			+	+
PB2:	WT	F			WT	F

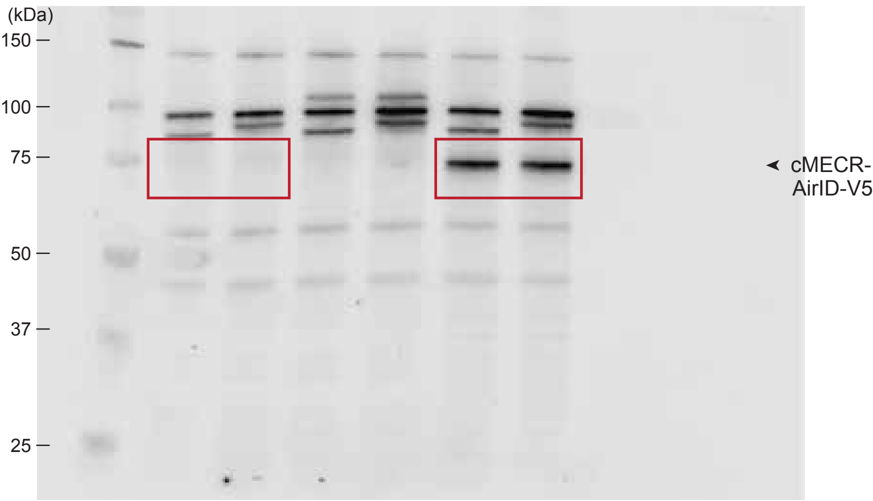

Fig. S5f (right)

Input, Western blot, α-PB1/PB2, chemiluminescence, Odyssey Fc

PB1/PA:	+	+	X	X	+	+
cMECR-AirID:	-	-			+	+
PB2:	WT	F			WT	F

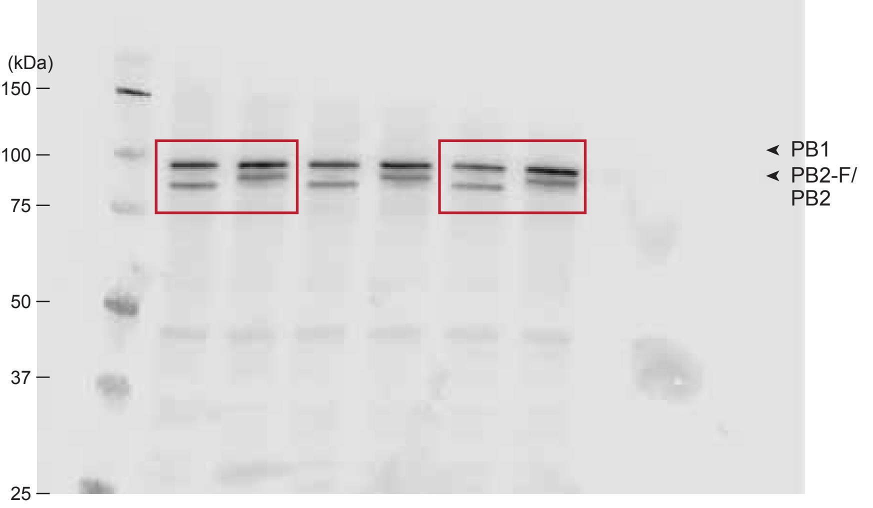

Fig. S5f (right)

Input, Western blot, α-SA, chemiluminescence, Odyssey Fc

PB1/PA:	+	+	X	X	+	+
cMECR-AirID:	-	-			+	+
PB2:	WT	F			WT	F

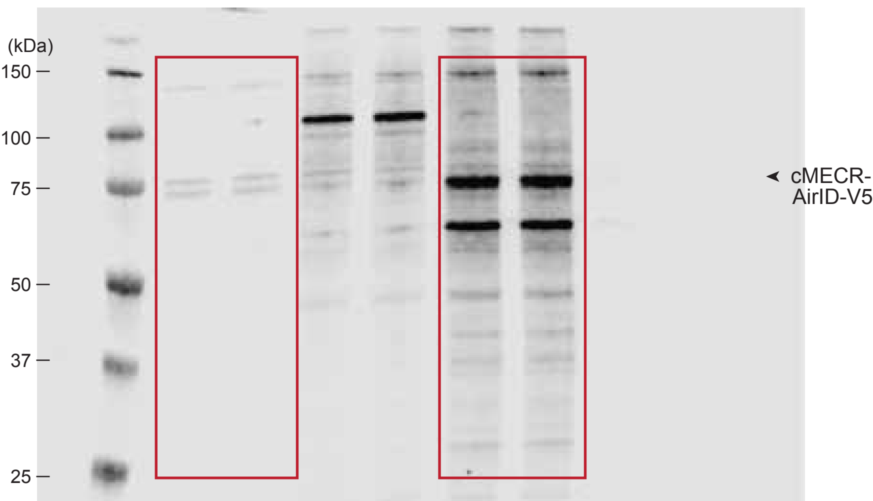

Supplement: S1 Raw Images — Areas of interest used in figures are outlined with a red box. Experimental approach, antibody (where relevant), detection strategy, and equipment are indicated for each image. Sizes are shown corresponding to molecular weight markers for proteins or 100 bp ladders for PCR products. Lanes not used in the figure are marked with “X.” (PDF) [file pbio.3001934.s011.pdf]
