# Supplementary material for: Alternative splicing liberates a cryptic cytoplasmic isoform of mitochondrial MECR that antagonizes influenza virus
Source: PLoS Biol. 2022 Dec 21;20(12):e3001934. doi: 10.1371/journal.pbio.3001934 (PMC9815647; doi:10.1371/journal.pbio.3001934)
Supplement: Attachment — Submitted filename: Rebuttal Letter.docx [file pbio.3001934.s013.docx]

Based on our Academic Editor's assessment of your work, the previous reviewers' reports and the revised manuscript, we are likely to accept this manuscript for publication, provided you clearly state and discuss the following limitations. As raised by reviewer #1, you never show that the endogenous cMECR is produced and responsible for inhibition of influenza virus and the evidence that cMECR restricts influenza viruses under physiological conditions is not compelling. Similarly, the concept of embedding an antiviral activity in an essential function is interesting but not fully convincing in this case. You show that siRNA silencing of MECR increases virus replication. Thus, the virus should still be able to counteract this antiviral mechanism by reducing MECR and cMECR expression levels or by specific targeting of cMECR in the cytoplasm. Although whether endogenous cMECR indeed restricts IAV in infected cells remains to be clearly demonstrated, we think that the manuscript contains a lot of suggestive evidence for an interesting inhibitory mechanism. Ideally, to address this concern, presenting direct evidence for endogenous cMECR expression at levels sufficient for inhibition would greatly enhance the significance of the study. However, you certainly already tried hard to achieve this without success during the revision process. Therefore, it is very important to modify the abstract and the rest of the manuscript accordingly to clearly state and discuss the limitations of the work.

We agree and appreciate the opportunity to highlight the strengths and limitations for the reader. We have edited the manuscript to tone down conclusions and draw attention to limitations. Specifically:

- in the abstract, the final paragraph of the introduction, and the first paragraph of the introduction, we now make it clear that functional data were generated when cMECR was ectopically expressed. Similarly, we soften the major conclusion by eliminating definitive claims and instead indicating that our data support a model where cMECR is embedded in MECR.
- we highlight limitations of our data:
  - line 288-291 indicate “These data suggest translation of cMECR. However, cMECR is a minor variant and differs from MECR only by a small change in molecular weight, preventing us from unambiguously detecting endogenous cMECR by western blot.”
  - conclusion on line 300-302 is softened.
  - minor changes on lines 384-387 also conditionalize our results
  - We include new text to clearly state limitations of our data. The end of the discussion has also been heavily edited. We include a separate paragraph discussing the limitations of our study, how we attempted to address them, and the remaining unknowns. We also narrow the scope of our final concluding paragraph. These are on lines 457-482 and copied here for convenience:

*A limitation of our study is the inability to uniquely detect endogenous cMECR, and as a consequence, to test cMECR function when natively expressed. cMECR is a minor variant and differs from MECR only by the absence of the first 76 amino acids. Smaller protein products were detected by blotting with antibodies targeting MECR (****Fig. 4e****), but these were nonspecific as they were also present in both of our MECR^-/-^ cell lines. Even if these were specifically detected, it would challenging to differentiate whether these smaller fragments arose from translation of cMECR or from proteolytic processing of full-length MECR. We have used bioinformatic analysis, splicing assays and genetic approaches to address this limitation. RNA-seq identified splice isoforms that code for cMECR, and ribosome profiling provided support for translation initiation at the cMECR start codon (****Fig. 5a*** *and* ***S6c****). Reporter assays showed that splicing of a model MECR transcript enabled initiation at the cMECR start codon (****Fig 5b****). Finally, ectopically expressing cMECR, but not MECR, was sufficient to confer an antiviral phenotype (****Fig. 6a, f****). Nonetheless, the full antiviral activity of endogenous cMECR remains to be clearly demonstrated.*

*Viruses often exploit conserved essential host genes whose critical role for the cell limits mutational escape. Conversely, host antiviral genes are often nonessential and can undergo mutation or regulated expression to thwart infection and limit self damage. Our study provides an example of a gene that is both essential and antiviral. Embedding the antiviral cMECR within the essential metabolic enzyme MECR establishes a Corneillian dilemma for influenza virus. Viral antagonism of cMECR would require discrete targeting of the cytoplasmic isoform and could have the off-target effect of disabling MECR and mtFAS, a metabolic process important for cell viability and influenza virus output. Conversely, leaving MECR and mtFAS intact would ensure cell health, while the virus would remain vulnerable to cMECR. We suggest that the host strategy of encoding an alternatively spliced antiviral protein that shares structure with an energetically important protein may represent a failsafe for the host, forcing the virus to make the difficult “decision” of antiviral antagonism or maximum biosynthetic power.*

Please also make sure to address the following data and other policy-related requests.

1. DATA POLICY:

A) Supplementary files (e.g., excel). Please ensure that all data files are uploaded as 'Supporting Information' and are invariably referred to (in the manuscript, figure legends, and the Description field when uploading your files) using the following format verbatim: S1 Data, S2 Data, etc. Multiple panels of a single or even several figures can be included as multiple sheets in one excel file that is saved using exactly the following convention: S1_Data.xlsx (using an underscore).

We now included S1_Data.xlsx with a separate tab for each panel.

B) Deposition in a publicly available repository. Please also provide the accession code or a reviewer link so that we may view your data before publication.

Our manuscript already contains the accession code for our RNASeq (PRJNA667475). This is now also reported in the Data Statement.

Regardless of the method selected, please ensure that you provide the individual numerical values that underlie the summary data displayed in the following figure panels as they are essential for readers to assess your analysis and to reproduce it: Figures 1CD, 2ABCD, 3B, 4BCEF, 6ADEF, and Supplementary Figures S1AB, S2ABCD, S3A, S6BC.

NOTE: the numerical data provided should include all replicates AND the way in which the plotted mean and errors were derived (it should not present only the mean/average values).

**Please also ensure that figure legends in your manuscript include information on where the underlying data can be found, and ensure your supplemental data file/s has a legend.**

We have added text to the end of all Figure Legends and Supplemental Figure Legends indicating where individual data and uncropped images can be found.

**Please ensure that your Data Statement in the submission system accurately describes where your data can be found.**

We have edited the Data Statement to point to raw data files in Supporting Information and deposited data sets.

2. BLOT AND GEL REPORTING REQUIREMENTS:

We require the original, uncropped and minimally adjusted images supporting all blot and gel results reported in an article's figures or Supporting Information files. We will require these files before a manuscript can be accepted so please prepare and upload them now. We require this for Figures 1B, 2C, 3ACD, 4DE, 5CDE, 6BCF, and Supplementary Figure S5BDF.

Please carefully read our guidelines for how to prepare and upload this data: <https://journals.plos.org/plosbiology/s/figures#loc-blot-and-gel-reporting-requirements>

We have uploaded raw uncropped images collated in S1_Raw_Images.pdf

As you address these items, please take this last chance to review your reference list to ensure that it is complete and correct. If you have cited papers that have been retracted, please include the rationale for doing so in the manuscript text, or remove these references and replace them with relevant current references. Any changes to the reference list should be mentioned in the cover letter that accompanies your revised manuscript.

Done. Citations updated as needed.

We expect to receive your revised manuscript within two weeks. Please, let me know if you need more time. 
